# Cross‐Level Regulatory Interactions Underlying Human Immune Aging

**DOI:** 10.1002/mco2.70817

**Published:** 2026-06-15

**Authors:** Quanyou Wu, Botao Zhang, Xiaochen Zhi, Qi Zhang, Kai Zhang, Yaru Wang, Kaitai Zhang, Lin Feng, Shujun Cheng, Ting Xiao

**Affiliations:** ^1^ State Key Laboratory of Molecular Oncology Department of Etiology and Carcinogenesis National Cancer Center/National Clinical Research Center for Cancer/Cancer Hospital Chinese Academy of Medical Sciences and Peking Union Medical College Beijing China; ^2^ Division of Abdominal Cancer Department of Medical Oncology Cancer Center and Laboratory of Molecular Targeted Therapy in Oncology West China Hospital Sichuan University Chengdu China; ^3^ Department of Neuro‐oncology Cancer Center Beijing Tiantan Hospital Capital Medical University Beijing China; ^4^ Department of Cancer Prevention National Cancer Center/National Clinical Research Center for Cancer/Cancer Hospital Chinese Academy of Medical Sciences and Peking Union Medical College Beijing China

**Keywords:** aging, immune, molecular interactions, multi‐omics, regulatory networks

## Abstract

A comprehensive understanding of age‐related changes in the human immune system is critical for deciphering the mechanisms of immunosenescence. Although transcriptomic studies have described immune alterations during aging, investigations into the proteomic layer and integrated multi‐omics approaches remain scarce. Here, we performed multi‐omics sequencing on peripheral blood samples from 69 individuals aged 23–75 years. We identified widespread dysregulation of RNA splicing in aging immune cells, with exon skipping being the most prevalent splicing event, which was mainly enriched in protein regulation‐related processes. Notably, lncRNAs such as SNHG1 and RP1‐3J17.3 modulated the expression of splicing‐regulatory mRNAs and proteins in aging immune cells, thereby influencing the RNA splicing of downstream genes. Splicing dysregulation of genes including EIF4G1, a translation initiation factor, led to alterations in protein translation, modification, and degradation, ultimately reshaping protein profiles in aging leukocytes. These proteomic alterations in our data reflect a gradual weakening of T‐cell function during aging. Together, unlike previous studies that focused only on single molecular layers, our study elucidates the interconnected nature of molecular regulation within leukocytes during aging, uncovers cross‐level interactions and regulatory networks, and provides a comprehensive multi‐omics resource for understanding immune aging and identifying potential strategies to delay immunosenescence.

## Graphical abstract

We performed multi‐omics sequencing on peripheral blood samples from 69 individuals aged 23–75 years and identified multi‐layered molecular interaction networks underlying the process of immune senescence, which provides a comprehensive multi‐omics framework for understanding immune aging and identifying potential strategies to delay immunosenescence.

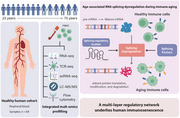

## 1 Introduction

Aging is a multifaceted biological process characterized by progressive physiological and molecular alterations that are closely associated with increased morbidity, organ dysfunction, and mortality [1, 2]. This intricate process significantly influences the immune system, instigating modifications in immune function and subtypes. As time progresses, the immune system experiences a gradual decline in its capacity to respond to foreign pathogens and infections, coupled with a diminished ability to recall and refine memory responses [3, 4]. Concurrently, circulating inflammatory mediators often increase moderately, a phenomenon termed “inflammaging” [5, 6]. Thus, immune aging is characterized by impaired host defense and an increased burden of chronic inflammatory diseases.

A comprehensive characterization of age‐related alterations in the human immune system is imperative for comprehending the molecular mechanisms underlying immunosenescence, elucidating the intricate connections between immune dysregulation and disease susceptibility in older individuals. In parallel, rapid advances in multi‐omics technologies have substantially expanded our ability to interrogate immune aging with enhanced resolution and functional depth. To date, most studies of immune senescence have predominantly relied on transcriptomic approaches [7, 8, 9]; however, transcriptomic profiling alone is insufficient to capture post‐transcriptional regulation, protein abundance, and functional proteoforms that ultimately govern cellular behavior [10, 11, 12].

Recent studies indicate that immune aging is driven not only by transcriptional reprogramming but also by extensive post‐transcriptional and proteomic remodeling, particularly age‐associated alternative splicing and proteoform diversity, which reshape immune function and antigenic landscapes [13, 14, 15, 16]. In support of this concept, a recent large‐scale proteomics resource constructed the first comprehensive atlas of protein isoforms generated by alternative splicing, revealing numerous non‐canonical proteoforms implicated in biological pathways relevant to aging and neurodegeneration [17]. Complementarily, growing evidence highlights the impact of aging on alternative splicing regulation: a recent study demonstrated widespread age‐dependent splicing alterations in human peripheral blood, some of which give rise to novel peptide sequences that were experimentally validated at the protein level, suggesting previously unrecognized mechanisms of age‐related immune regulation [18]. Together, these findings indicate that single‐layer transcriptomic analyses are insufficient to capture the functional complexity of immunosenescence and underscore the need for integrative multi‐omics‐ approaches. Despite these advances, studies simultaneously profiling matched transcriptomic, proteomic, and immune repertoire layers across age groups remain scarce, although such approaches can provide valuable, non‐redundant biological insights [19].

In this study, we collected peripheral blood samples from individuals across different age groups and conducted comprehensive profiling of transcriptomes, proteomes, and T‐cell receptor (TCR) repertoires within the same set of samples. In addition, publicly available single‐cell sequencing datasets were incorporated to enhance cellular resolution. By integrating multi omic layers, our analysis provides novel insights into how aging reshapes immune composition and function, enabling a deeper understanding of immunosenescence.

## 2 Results

### 2.1 Changes in Immune Cell Abundance and mRNA Expression With Aging

**FIGURE 1:**
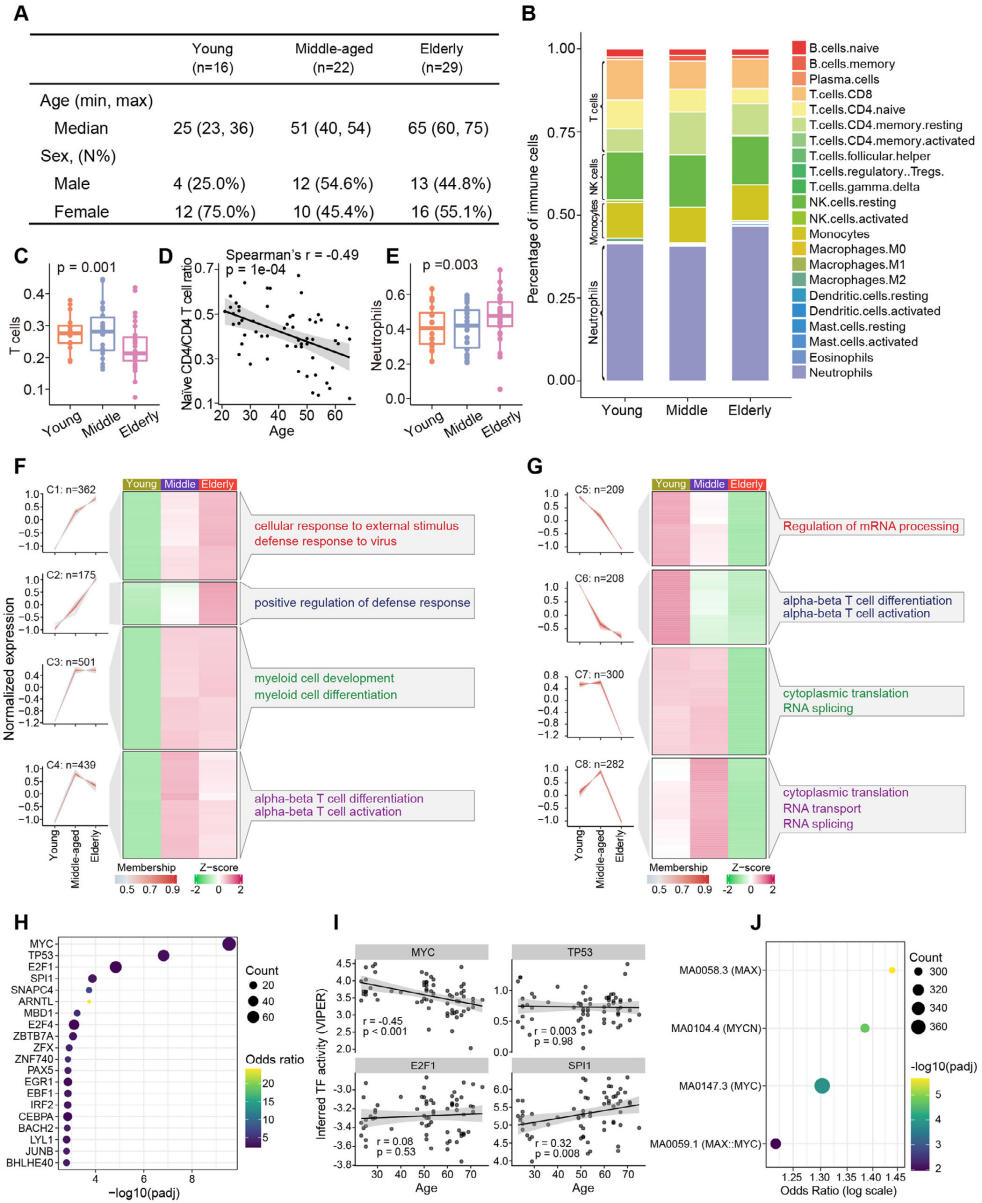
Changes in immune cell abundance and mRNA expression with aging. (A) Grouping information statistics for transcriptome sequencing samples in the aging cohort. (B) Barplot illustrating the proportions of immune cells in the three groups. (C) Boxplot showing the proportions of T cells in the three groups. (D) Dot plots illustrating the correlation between age and the ratio of naïve CD4 T cells to total CD4 T cells. (E) Boxplot showing the proportions of neutrophils in the three groups. (F and G) The leftmost line graph and the middle heatmap depict the expression trends of these genes across the three age groups, with the rightmost text indicating the enriched GO terms for the respective gene sets. (H) Dot plot of TF regulon enrichment among age‐associated mRNAs. Each point represents a TF regulon (DoRothEA, confidences A–C); point size indicates the number of overlapping target genes and color denotes odds ratio. (I) Inferred sample‐level TF activities (VIPER) and their associations with age. (J) Promoter motif enrichment analysis of age‐associated genes. The dot plot displays significantly enriched motifs, with dot color indicating the odds ratio and dot size representing the number of age‐associated genes containing the motif. Motifs annotated as MAX, MYCN, MYC, and the MAX::MYC heterodimer were significantly enriched.

We performed RNA sequencing on peripheral blood samples from 67 subjects, classified as young (*n* = 16), middle‐aged (*n* = 22), and elderly (*n* = 29) (Figure 1A; Table S1). To evaluate the potential confounding effect of the extended sample collection period, we performed a principal component analysis on the global transcriptomic profiles. The analysis revealed that samples did not cluster by collection year. Instead, the major source of molecular variation was distinctly stratified by age (Figure S1A,B), indicating that technical and seasonal effects were negligible relative to aging‐associated biological signals. Using CIBERSORT on peripheral white blood cell mRNA profiles, we estimated the distribution of 22 immune cell subtypes. This analysis revealed an overall decrease in T cells with advancing age, especially the naïve CD4 T cells (Figure 1B,C), which are essential for adaptive immune initiation, antigen recognition, and immune tolerance. Because age‐related reductions in naïve CD4 T cells have not been consistently reported [20, 21, 22], we validated this finding in an independent cohort (*n* = 58; age range, 21–65 years) using flow cytometry. The results confirmed a consistent age‐associated decline in naïve CD4 T‐cell proportions (Figure 1D; Table S2). We also observed an upward trend in the proportion of neutrophils with age (Figure 1B,E), whereas no significant differences were detected in the proportions of other immune cells, such as monocytes (MC), B cells, and NK cells (Figure 1B). These findings align with results obtained from previous single‐cell level analyses [9], underscoring the reliability of our transcriptomic data.

Through the fuzzy c‐means algorithm from “ClusterGVis” R package, we observed that protein‐coding mRNAs exhibiting an age‐related increase in expression can be categorized into four types: C1, a rapid increase from young to middle age followed by a slower increase in old age; C2, a continuous rapid increase across age groups; C3, a rapid increase from young to middle age followed by stable expression in old age; and C4, a rapid increase from young to middle age followed by decline in old age (Figure 1F). Notably, the genes in C1 and C2, the two sets with continuous upregulation across young, middle‐aged, and old age, are predominantly enriched in the “defense response to external stimulus.” The genes in C3 are mainly enriched in “myeloid cell development and differentiation,” indicating increased activity of myeloid cells in middle age compared to young age, which is maintained in old age. On the other hand, the genes in C4 are primarily enriched in “alpha‐beta T cell activation and differentiation,” suggesting a gradual decline in the functionality of alpha‐beta T cells from middle age to old age (Figure 1F).

Among protein‐coding genes exhibiting a decline in expression with age, there are also four distinct types: C5, a rapid decrease from young to middle age followed by an accelerated decline in old age; C6, a rapid decrease from young to middle age followed by slower decline; C7, stable expression from young to middle age followed by marked decline in old age; and C8, increased expression from young to middle age followed by accelerated decline in old age (Figure 1G). Notably, C6 is predominantly enriched in “alpha‐beta T cell activation and differentiation,” corroborating the observation in C4 and collectively indicating a gradual decline in the functionality of alpha‐beta T cells from middle age to old age. Genes in C5, C7, and C8 are commonly enriched in “mRNA processing, RNA splicing, and cytoplasmic translation,” suggesting a rapid decrease in the expression of genes related to RNA splicing and protein translation from middle age to old age (Figure 1G).

Transcription factor (TF) regulatory programs are associated with age in peripheral blood leukocytes. Regulon enrichment analysis showed that targets of several TFs were significantly overrepresented among age‐associated mRNAs, with MYC, TP53, E2F1, and SPI1 among the top enriched TFs (Figure 1H). To investigate whether TF activity itself changes with age, we inferred sample‐level TF activities from the bulk RNA expression data using VIPER with DoRothEA regulons. MYC activity across samples showed a significant negative association with age, indicating a decline of inferred MYC activity in older individuals. In contrast, SPI1 activity increased with age. TP53 and E2F1 activities did not show consistent correlation with age in this cohort (Figure 1I). To further assess evidence of direct DNA binding, we scanned promoters (–2 kb to +200 bp relative to transcription start site) of the age‐associated genes for known TF motifs (JASPAR PWMs). Promoter motif enrichment analysis revealed significant enrichment of motifs annotated as MAX, MYCN, MYC, and MAX::MYC heterodimer within promoters of age‐associated genes (Figure 1J). These factors belong to the basic helix–loop–helix leucine zipper (bHLH‐LZ) family that recognize canonical E‐box sequences (e.g., CACGTG) and regulate programs of cell growth, metabolism, ribosome biogenesis, and cell‐cycle progression. MYC (commonly referred to as c‐Myc) and MYCN (N‐Myc) act as transcriptional activators when dimerized with the obligate partner MAX. In peripheral blood leukocytes, MYC–MAX activity is tightly linked to cellular activation and proliferative responses [23]. Taken together, these results implicate the MYC–MAX axis in regulation of age‐associated transcriptional programs.

### 2.2 lncRNAs Mediate Immunoinflammatory State and Splicing Dysregulation During Aging by Modulating mRNA

**FIGURE 2:**
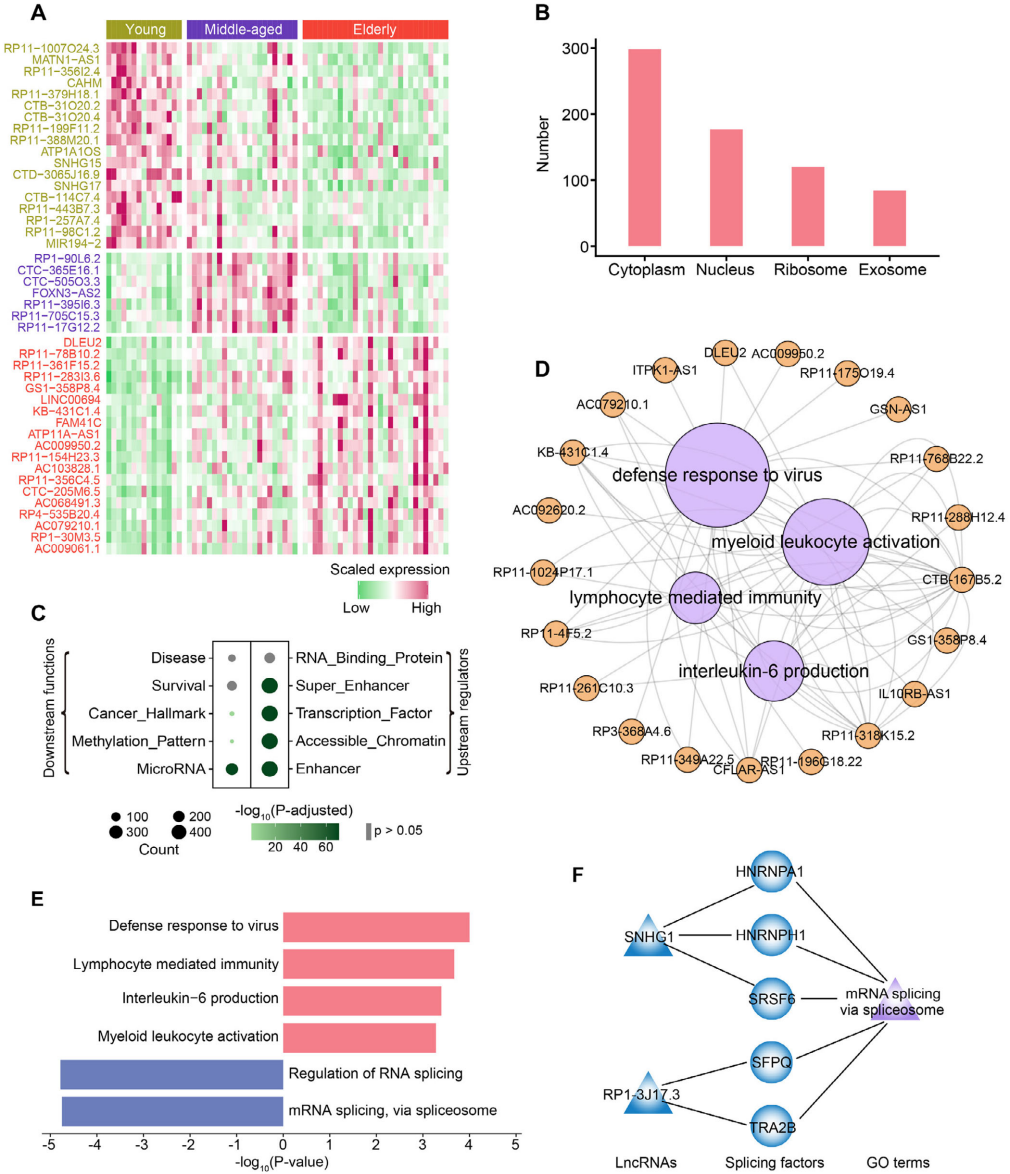
LncRNAs mediate immunoinflammatory state and splicing dysregulation during aging by modulating mRNA. (A) Heatmap showing the most highly expressed lncRNAs in each age group. Red indicates high expression while green represents low expression. (B) Bar chart illustrating the subcellular localization distribution of age‐associated lncRNAs. (C) The left half of the dot plot displays the downstream effects of age‐associated lncRNAs, while the right half shows the factors regulating age‐associated lncRNAs. Gray dots represent *p* > 0.05, and the color intensity of the dots indicates the significance level. Larger dots denote a higher abundance of age‐associated lncRNAs in the corresponding category. (D) lncRNA‐mRNA co‐expression regulatory network. Orange circles indicate upregulated lncRNAs with age. The central purple circle represents the GO enrichment results of mRNAs co‐expressed with these lncRNAs. The size of purple circles indicates the number of co‐expressed mRNAs in each Gene Ontology term. (E) Enrichment results of mRNAs co‐expressed with age‐associated lncRNAs. Pink bars represent the enrichment results of mRNAs co‐expressed with upregulated lncRNAs with age, while blue bars represent the enrichment results of mRNAs co‐expressed with downregulated lncRNAs with age. (F) Regulatory networks summarizing the lncRNA‐splicing factor correlation pairs, where blue triangles indicate downregulated lncRNAs with age, and blue circles represent co‐expressed splicing factors.

Presently, it is well established that lncRNAs play pivotal roles in various biological processes, and several have been implicated in aging. To systematically characterize lncRNA changes during aging, we analyzed our expression profiles and detected 2428 lncRNAs, of which 560 were age‐associated, including 300 upregulated and 260 downregulated lncRNAs (Table S3). The most highly expressed lncRNAs in each age group are illustrated in Figure 2A. Particularly, lncRNAs highly expressed in the elderly group may serve as potential biomarkers of immune aging. We further observed that age‐associated lncRNAs are primarily localized in the cytoplasm (Figure 2B). Cytoplasmic lncRNAs are often involved in post‐transcriptional regulation and impact processes such as mRNA stability, protein translation, and the competitive endogenous RNA (ceRNA) network. Further functional enrichment analysis using LncSEA [24] showed that these lncRNAs primarily regulate miRNAs (Figure 2C), thereby influencing mRNA expression and supporting their predominant cytoplasmic function. Their expression was mainly regulated by enhancers, TFs, and accessible chromatin, rather than RNA‐binding proteins (Figure 2C).

Further analysis revealed that 201 mRNAs were co‐expressed (correlation coefficient > 0.85) with the upregulated lncRNAs associated with aging, predominantly enriching in defense response to virus and myeloid leukocyte activation (Figure 2D,E). Literature reports indicate that aging of the immune system is characterized by sustained low‐level activation of innate immune cells, leading to an inflammatory environment, which may be correlated with lncRNAs. Moreover, only 20 mRNAs were co‐expressed (correlation coefficient > 0.85) with the downregulated lncRNAs over time. Notably, five of these mRNAs are splicing factors, exhibiting significant enrichment in RNA splicing. These splicing factors were mainly co‐expressed with SNHG1 and RP1‐3J17.3 (Figure 2E,F). This suggests that the rapid decline in the expression levels of RNA splicing‐related genes from middle age to old age is, in part, attributable to lncRNA.

### 2.3 Age‐Associated Changes in mRNA Expression of Immune Cells Revealed by Single‐Cell Transcriptomics

**FIGURE 3:**
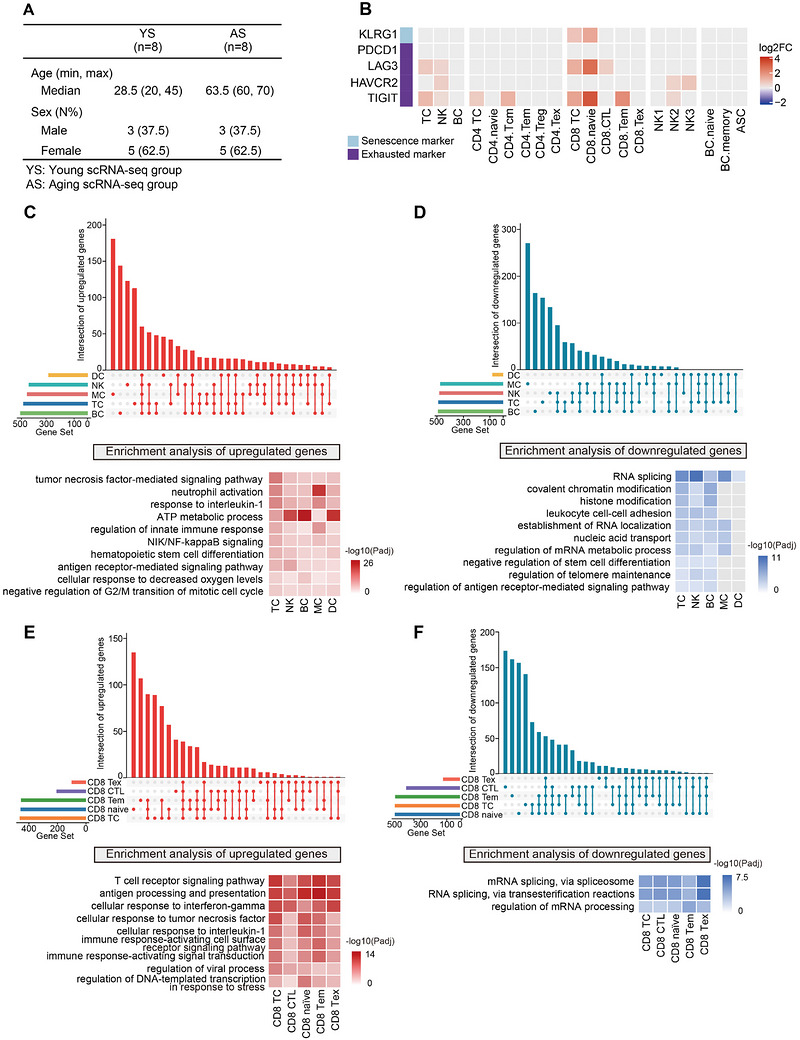
Age‐associated changes in mRNA expression of immune cells revealed by single‐cell transcriptomics. (A) Statistical information on sample grouping in single‐cell transcriptome sequencing. (B) Expression characteristics of molecular markers associated with cell aging and exhaustion across different cell subtypes. Red indicates high expression in the elderly group, while blue represents low expression in the elderly group. (C and D) The upper panel displays an UpSet plot depicting the intersection of genes upregulated (C) and downregulated (D) in the five major immune cell types during aging. The heatmap below illustrates the biological function enrichment results of these genes. (E and F) The upper panel displays an UpSet plot depicting the intersection of genes upregulated (E) and downregulated (F) in various CD8 T‐cell subtypes during aging. The heatmap below illustrates the biological function enrichment results of these genes.

The preceding bulk RNA‐seq analysis of mixed immune cells provided an overview of aging‐related features in the peripheral immune system. To further examine immune cell subtype‐specific aging signatures, we analyzed a single‐cell transcriptomic dataset of PBMCs from 16 healthy individuals stratified by age (Figure 3A).

Using canonical immune cell markers and immune cell‐enriched gene sets, t‐SNE clustering identified five major immune cell types: T cells (TC), natural killer cells (NK), B lymphocytes (BC), MC, and dendritic cells (DC) (Figure S2A). CD3 T cells were further re‐clustered into CD4 and CD8 T cells. CD4 T cells were subdivided into naïve CD4 cells, central memory T cells (Tcm), effector memory T cells (Tem), regulatory T cells (Treg), and exhausted T cells (Tex), whereas CD8 T cells were subdivided into naïve CD8 cells, cytotoxic T lymphocytes, CD8 Tem, and CD8 Tex (Figure S2B,C). NK cells were re‐clustered into immune regulatory NK1, cytotoxic NK2, and late‐stage NK3 cells (Figure S2D). B lymphocytes were divided into naïve B cells, memory B cells, antibody‐secreting cells (ASC) with high MZB1 expression, and age‐associated B cells (ABCs) with high ITGAX expression (Figure S2E). We then systematically analyzed aging features across these major immune cell classes and subtypes.

We first constructed a differentially expressed gene set associated with aging (adjusted *p*‐value < 0.05, |Log2FC| > 0.25). At the single‐cell level, specific aging signals were observed (Figure S3A), such as the upregulation of classical cellular aging markers CDKN1A (p21) and CDKN2A (p16) across various immune cell types. Concerning lymphocytes, we assessed the expression levels of several lymphocyte‐specific aging and exhaustion biomarkers. The results revealed varying degrees of immune aging‐associated alterations among different lymphocyte subgroups. Among the five immune cell types, T lymphocytes exhibited the most pronounced abnormal changes, with CD8 T‐cell subtypes showing the most significant age‐related gene expression alterations (Figure 3B). Both T lymphocytes and NK lymphocytes displayed varying degrees of exhaustion characteristics (Figure 3B).

Indeed, beyond aging and exhaustion markers, each cell subtype exhibited broad transcriptomic remodeling during aging. We conducted separate analyses for the differential genes in each of the five major immune cell types and their respective immune cell subtypes. Overall, both lymphoid cells (TC, BC, NK) and myeloid cells (MC, DC) exhibit pronounced changes in their transcriptomes with aging. These cells consistently upregulate signaling pathways mediated by TNF, myeloid cell activation, innate immune response, and the G2/M phase transition of the cell cycle, while processes such as RNA splicing, nucleic acid transport and localization, histone modification, and telomere maintenance show widespread downregulation (Figure 3C,D). Subsequently, we delved into the distinctive biological processes associated with the dysregulation of various lymphocyte subtypes. Focusing on CD8 T cells, the upregulated genes are not only involved in TNF‐mediated immune response but also participate in IL**‐**1 response, TCR signaling pathways, and antiviral regulation. Conversely, the downregulated genes are significantly enriched in biological processes such as RNA splicing (Figure 3E,F). CD4 T cells showed similar upregulated biological processes (Figure S4A), but downregulation of T‐cell activation, differentiation, and cell adhesion suggested impaired immune regulatory function (Figure S4B). Notably, the significant upregulation of cytotoxic negative regulation processes in NK cells, coupled with the downregulation of B‐cell proliferation and activation, as well as B‐cell receptor‐mediated signaling pathways, collectively indicates a decline in the antiviral activity of these cells (Figure S4C–F). It is noteworthy that RNA splicing‐related genes are downregulated with increasing age in both NK cell subtypes and B‐cell subtypes (Figure S4D,F). Thus, single‐cell transcriptomics confirmed bulk transcriptomic findings, including peripheral immunoinflammatory activation and RNA splicing dysregulation, while revealing subtype‐specific immune aging signatures.

### 2.4 Protein Expression Dynamics and Their Interaction With lncRNA and RNA Splicing During Aging

**FIGURE 4:**
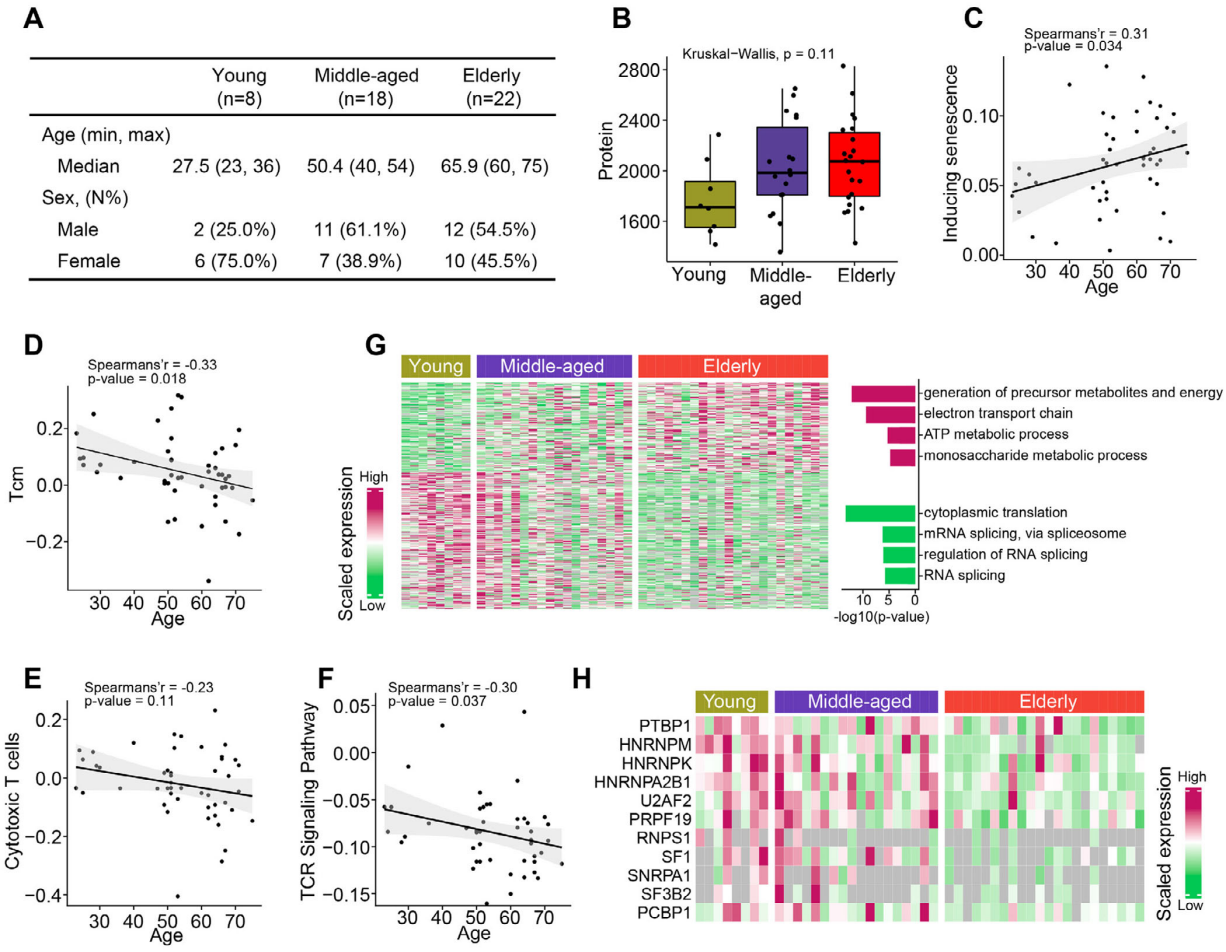
Protein expression dynamics and its interaction with lncRNA and RNA splicing through aging. (A) Statistical information on sample grouping in mass spectrometry. (B) Box plot showing the number of identified proteins in each sample within the three age groups. (C) Scatter plot demonstrating the overall increase in the expression of pro‐aging proteins with age. (D–F) Scatter plots illustrating the decreased activity of Tcm, cytotoxic T cells, and TCR signaling pathway with increasing age. (G) The left half of the figure presents a heatmap of proteins whose expression increases and decreases with age, while the red bars on the right represent the enrichment analysis results for proteins upregulated with age, and the green bars represent the enrichment analysis results for proteins downregulated with age. (H) Heatmap depicting the expression levels of splicing factors identified by proteomics that are downregulated with age.

We selected 48 samples from the aging cohort for mass spectrometry analysis (Table S1), with sample information shown in Figure 4A. Across all samples, 4344 proteins were identified, with slightly more proteins detected in the elderly group (Figure 4B, Figure S5A). Since all samples were processed on the same platform in randomized order, this may indicate that more proteins reached the mass spectrometry detection threshold in immune cells from elderly individuals. Before analysis, protein quantification values were corrected by total sum normalization, and distributions before and after correction are shown in Figure S5B,C. Among the 4344 proteins detected across the cohort, 130 were identified exclusively in the elderly group. Functional enrichment revealed that these proteins are predominantly associated with cell‐cycle regulation and DNA maintenance, including “cell cycle checkpoint signaling,” “DNA repair,” and “DNA damage checkpoint signaling” (Figure S5D). This enrichment suggests altered proliferative dynamics and an elevated engagement of DNA‐damage response pathways in immune cells from older individuals, which may reflect increased cellular stress, compensatory repair activity, or greater genomic instability within particular immune subpopulations.

Then, we performed a genome‐wide per‐gene correlation analysis between mRNA expression and protein abundance to characterize the concordance of transcriptomic and proteomic measurements. Correlation strength was classified based on the absolute correlation coefficient as follows: weak (0.1 ≤ |*r*| < 0.3), moderate (0.3 ≤ |*r*| < 0.5), and strong (|*r*| ≥ 0.5). Using this scheme, a total of 264 genes passed quality filters and were categorized: 10 (3.8%) showed weak correlation, 195 (73.9%) showed moderate correlation, and 59 (22.3%) showed strong correlation between mRNA and protein levels. Regarding directionality, 47 genes showed significant negative correlations, including four weak, 29 moderate, and 14 strong negative correlations. Among the strong negative set, PTPRA, TRIP12, DNAJC3, IGBP1, and TAP2 were the most statistically prominent genes ranked by absolute correlation magnitude (Figure S5E), suggesting substantial post‐transcriptional and/or translational regulation of their protein‐level changes.

We then conducted single‐sample gene set enrichment analysis on genes related to cellular senescence obtained from the CellAge database and observed a significant increase in pro‐aging‐related proteins within leukocytes as age advanced (Figure 4C). Subsequently, we aimed to assess the peripheral immune characteristics from the perspective of immune cell scoring based on our curated reference gene list (Table S4). We found that with increasing age, the immune scores of Tcm and cytotoxic T cells showed a decreasing trend (Figure 4D,E), and the TCR signaling pathway also exhibited a decreasing trend (Figure 4F). Therefore, the proteome reflects a gradual weakening of T‐cell function with age.

We further identified 349 proteins that undergo changes with age, including 139 proteins upregulated with age and 210 proteins downregulated with age (Table S5). Protein expression changes were significantly associated with lncRNA alterations: 67 age‐associated proteins strongly correlated with 225 age‐associated lncRNAs (|*r*| > 0.7). Age‐upregulated lncRNAs tended to be negatively correlated with age‐downregulated proteins, whereas age‐downregulated lncRNAs were mainly positively correlated with age‐downregulated proteins (Figure S6; Table S6). These findings suggest that proteins experiencing decreased expression with age may be influenced by changes in lncRNA expression. Among these proteins, HMGB1 and MECP2 are currently known to inhibit cellular senescence. Besides, the functions of proteins downregulated in response to lncRNA influence were primarily enriched in RNA splicing (Figure S6).

Among the 139 proteins upregulated with age, there was an enrichment in metabolism‐related genes, while the enrichment results for the 210 age‐associated proteins, which decreased with age, were primarily enriched in RNA splicing (Figure 4G). Eleven splicing factors were detected exhibiting downregulation with age in the proteomic analysis (Figure 4H). Hence, the bulk RNA‐seq, single‐cell transcriptome, and proteome consistently highlight RNA splicing as one of the markedly downregulated biological processes in leukocytes with age.

### 2.5 Alterations in RNA Splicing and Their Impact on Protein Profiles During Aging

**FIGURE 5:**
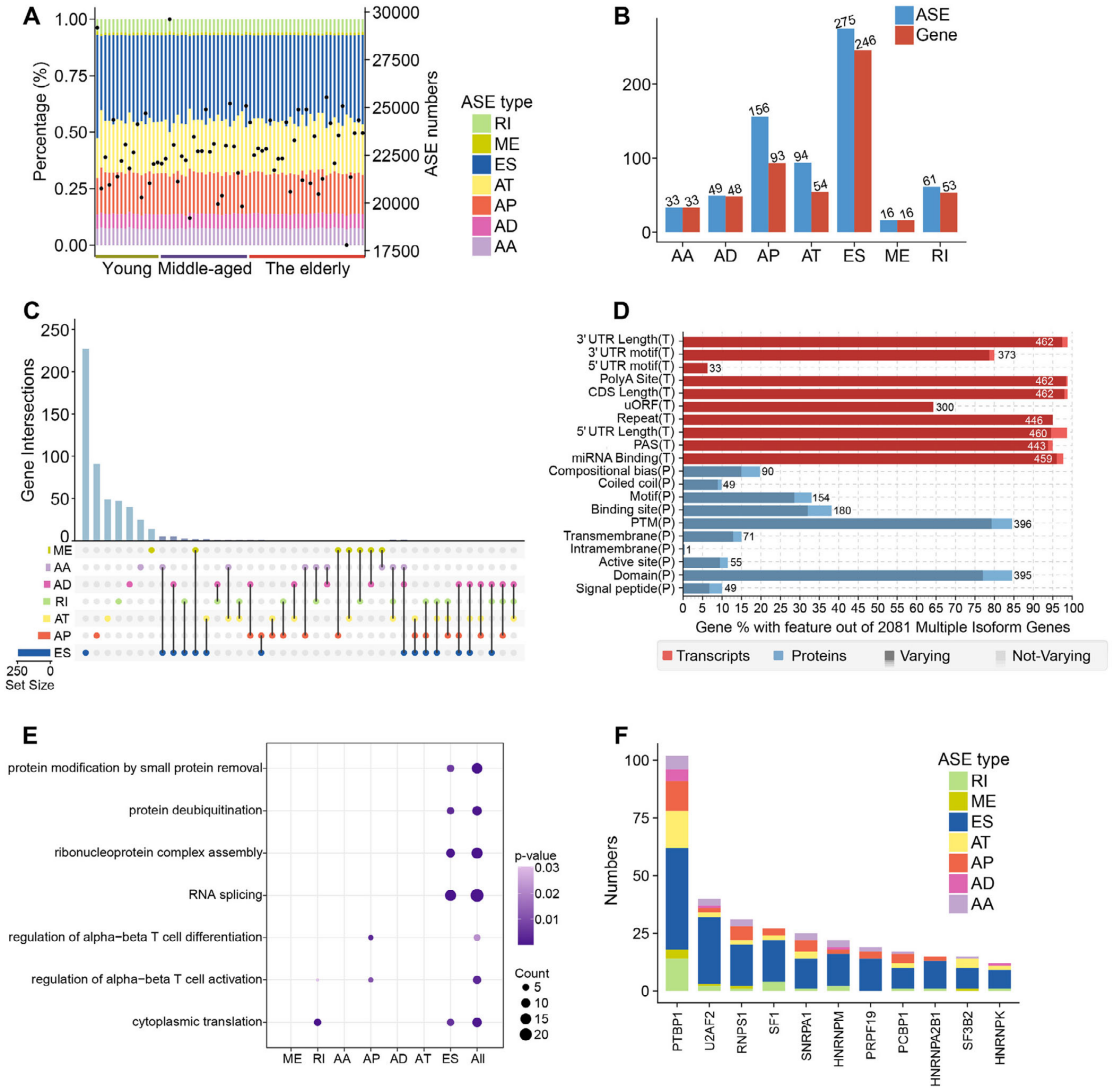
The alterations of RNA splicing and its impact on protein profiles through aging. (A) Overview of ASEs in peripheral blood samples from our aging cohort. Bars depict the proportion of the seven ASE types in each sample, and points indicate the total number of ASEs identified in each sample. (B) The count of the seven types of age‐associated ASEs and their associated genes identified in the aging cohort. (C) UpSet plot illustrating the intersections among the seven types of age‐associated ASEs. (D) The enumeration and proportion of genes exhibiting specific features (as indicated on the y‐axis) within the 517 genes influenced by age‐associated ASEs. The darker color in each bar represents the percentage of genes having isoforms that corresponding feature varies. (E) GO enrichment analysis results for genes affected by age‐associated ASEs, with darker colors indicating smaller p‐values and larger points representing a greater number of enriched genes. (F) The count of age‐associated ASEs regulated by each splicing factor downregulated with age.

We next examined age‐related RNA splicing changes in leukocytes. Seven alternative splicing event (ASE) types were identified: alternate acceptor site (AA), alternate donor site (AD), alternate promoter (AP), retained intron (RI), alternate terminator (AT), exon skipping (ES), and mutually exclusive exons (ME). Retaining only ASEs detected in at least two samples per group yielded 32,681 ASEs. The proportions of the seven ASE types were similar across age groups, and most samples contained >20,000 ASEs (Figure 5A). Correlation analysis identified 684 age‐associated ASEs, with ES being the most frequent, followed by AP (Table S7). These ASEs affected 517 genes (Figure 5B). Although some genes were affected by multiple ASE types, most were regulated by only one age‐associated ASE type (Figure 5C).

Among the 517 genes affected by age‐associated ASEs, most had isoforms with transcript‐level differences, including coding sequence (CDS) and 3′ untranslated region (UTR) length, as well as protein‐level differences, including protein domains and binding sites, suggesting that aberrant splicing may substantially alter gene function (Figure 5D). Known aging‐promoting genes, including AKT1, IRF5, and SREBF1, and aging‐inhibiting genes, including FOS, NEK1, RUVBL2, and SGK1, showed splicing alterations during aging (Table S7). Genes affected by RI, AP, and ES events were significantly enriched in biological processes. Age‐associated RI events primarily impacted “cytoplasmic translation,” AP events predominantly affected “regulation of alpha‐beta T cell activation and differentiation,” and while ES events mainly influenced RNA splicing and protein regulation‐related processes, such as “protein modification,” “protein deubiquitination,” and “cytoplasmic translation” (Figure 5E). ASEs in genes involved in protein translation, modification, and degradation may strongly influence the protein profile. Among the 4344 identified proteins, 2732 were strongly correlated with ASEs in these genes (|*r*| > 0.7; Table S8). Among 14,291 ASE–protein correlation pairs, ES events accounted for 51.1%. The ES event of CPSF7 showed strong correlations with 171 proteins, the largest number among all ASEs, followed by the ES event of EIF4G1, which correlated with 153 proteins (Table S8). These results suggest that splicing alterations in CPSF7, an mRNA processing factor, and EIF4G1, a translation initiation factor, may substantially affect protein expression profiles in aging immune cells. The 11 splicing factors previously identified as age‐downregulated in the proteome collectively regulated 325 age‐associated ASEs, with PTBP1 regulating the most ASEs (*n* = 102), followed by U2AF2 (*n* = 40) (Figure 5F).

To investigate whether alternative splicing events are reflected at the protein level, we used an isoform‐aware protein database from ENSEMBL and re‐searched the MS data to obtain isoform‐level quantification. Across all runs we identified 20,705 protein entries and obtained quantitative values for 4111 isoforms. However, only 224 genes had more than one quantified isoform, consistent with the well‐known limitation of shotgun proteomics that many transcript isoforms do not generate isoform‐unique tryptic peptides (or generate peptides that are too short or of too low abundance to be detected) [25, 26]. To avoid overinterpretation, we restricted isoform‐level claims to isoforms supported by isoform‐unique peptides. For these supported isoforms, we compared isoform intensities (log2 transformed and sample median‐centered) with transcript abundances [log2(TPM+1)] across 48 paired samples using Spearman correlation. Twelve transcript–protein isoform pairs showed positive associations (nominal *p* < 0.05 and Spearman *r* > 0.3) (Figure S7), indicating that ASEs are detectable as distinct isoforms in a subset of cases. Nevertheless, the limited number of multi‐isoform quantifications indicates that most ASEs cannot be unambiguously resolved at the protein level by standard discovery proteomics.

### 2.6 TCR Repertoire Landscape and Dynamics During Aging

TCRs recognize specific antigens through highly diverse antigen‐binding sites, enabling broad protection against external antigenic threats. To further characterize T cell function during aging, we analyzed TCR repertoire features in the aging cohort. TCRB libraries were constructed from peripheral blood samples of all 69 subjects using the SMART (Switching Mechanism At the 5' end of RNA Transcript) principle. Across samples, we detected 46 known V genes, 13 J genes, and 2 D genes constituting the TCRB chain.

**FIGURE 6:**
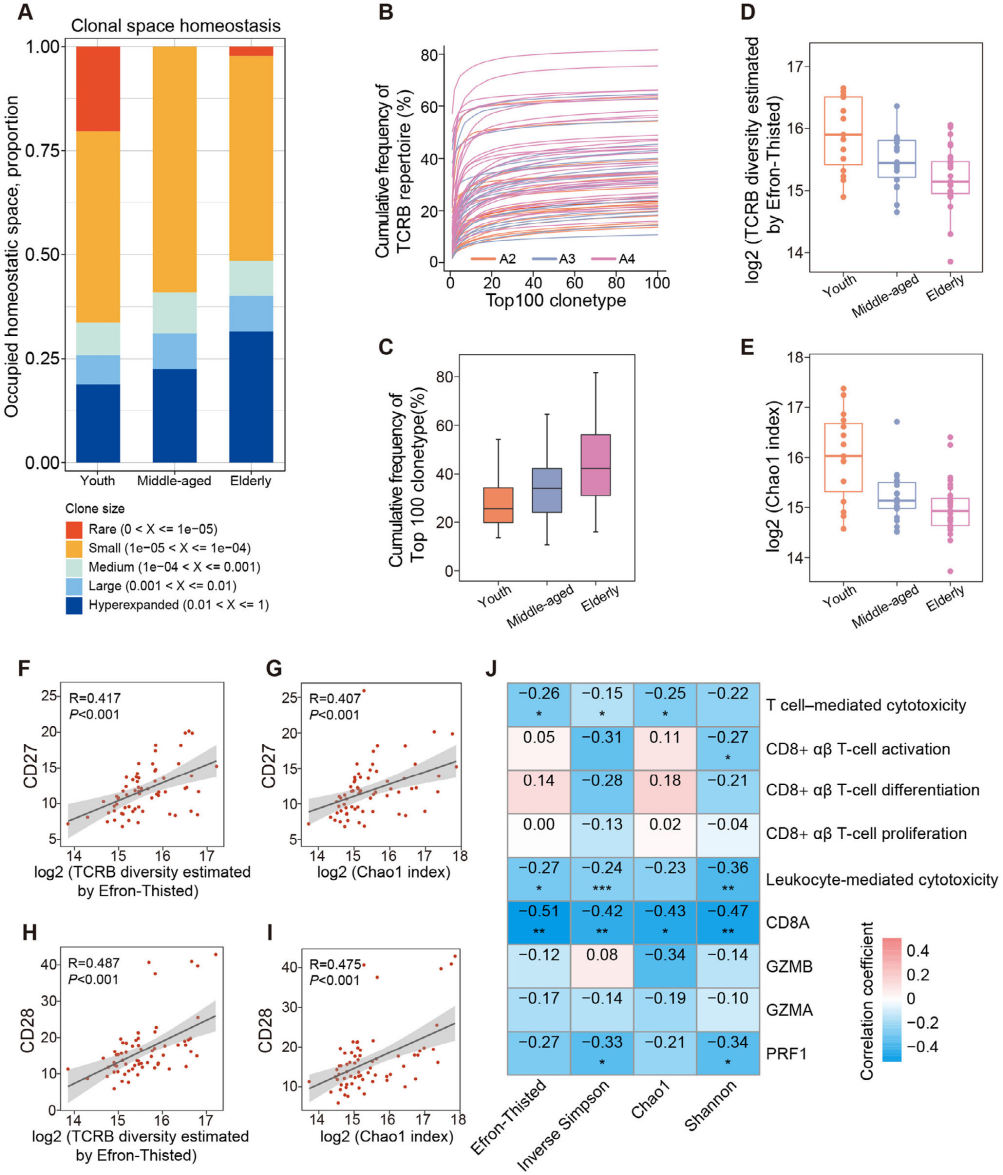
TCR repertoire landscape and dynamics through aging. (A) Bar chart illustrating the cumulative clone frequency of TCR at different clonal frequencies. (B) Cumulative frequency distribution of the top 100 clones. (C) Group comparisons of the cumulative frequency distribution of the top 100 clones. (D and E) Group comparisons of TCR repertoire diversity assessment metrics, including the Efron–Thisted index and Chao1 index. (F and G) Pearson correlation analysis between the expression level of T‐cell surface co‐stimulatory molecule CD27 and the two diversity evaluation metrics. (H and I) Pearson correlation analysis between the expression level of CD28 and the two diversity evaluation metrics. For optimized visualization, the numerical values of the two diversity metrics were log2‐transformed. (J) Heatmap showing Spearman correlation coefficients between TCR diversity metrics (columns) and ssGSEA gene‐set scores derived from RNA‐seq together with measured immune proteins from the proteomics dataset (rows). Cell colors represent the direction and magnitude of the correlations. Each cell is annotated with the corresponding Spearman correlation coefficient, and statistical significance is denoted by asterisks (***p < 0.001; **p < 0.01; *p < 0.05).

We observed a notable increase in the proportion of high‐frequency clones (clonal molecules with frequencies between 0.01 and 1) with advancing age in the population, while low‐frequency clones (clonal molecules with frequencies less than 0.00001) exhibited a pronounced decline with age (Figure 6A). In the middle‐aged group (around the age of 50), the proportion of low‐frequency clones had already reached a relatively low level. Similarly, we identified the top 100 clones based on the clonal frequencies in descending order and examined the cumulative clonal frequency characteristics. The results indicated that groups with older individuals had higher cumulative clonal frequencies for the top 100 clones (Figure 6B,C). Subsequently, we introduced two diversity indices, the Efron–Thisted diversity index and the Chao1 index, to assess the TCR diversity features in the aging body. The analysis revealed a gradual decrease in both diversity indices with increasing age, showing a high degree of consistency in the results (Figure 6D,E).

Reduced diversity indicates widespread clonal expansion in the TCR repertoire, which may impair recognition of diverse antigens. We further analyzed the relationship between TCR diversity and expression of the co‐stimulatory molecules CD27 and CD28, which are important for T‐cell activation and aging [27]. We identified a significant positive correlation between the expression of both molecules and the diversity assessment indices (Figure 6F–I). These findings indicate that aging is associated with reduced TCR repertoire diversity and decreased T‐cell co‐stimulatory signaling, potentially impairing normal T‐cell function.

To investigate whether changes in the TCR repertoire were reflected at the transcriptomic and proteomic levels, we correlated per‐sample TCR diversity indices (Efron–Thisted, Chao1, Shannon, and inverse Simpson) with ssGSEA module scores derived from RNA‐seq (cytotoxicity/activation/differentiation/proliferation gene sets) and with measured immune proteins in the proteomics dataset (e.g., CD8A, GZMA, GZMB, and PRF1). Notably, the vast majority of tested associations were negative, indicating that samples with lower TCR diversity tended to show higher transcriptional and proteomic cytotoxic signatures (Figure 6J). Representative examples include negative correlations between TCR diversity and cytotoxic signatures at both RNA and protein levels. The ssGSEA score for a T‐cell‐mediated cytotoxicity gene set was negatively associated with Efron–Thisted diversity, inverse Simpson, and Chao1. At the protein level, CD8A protein abundance was negatively correlated with multiple diversity metrics, and perforin (PRF1) protein showed similar negative associations.

These negative associations suggest that reduced repertoire diversity coincides with enhanced cytotoxic/effector signatures at the transcript and protein levels. This pattern is consistent with antigen‐driven clonal expansion of cytotoxic T cells: when a limited number of clones expand in response to persistent or recurrent antigenic stimulation, such as chronic inflammation, peripheral blood TCR diversity decreases while effector genes, including GZMB and PRF1, and CD8 markers increase because the expanded clones are predominantly cytotoxic. Thus, transcriptomic and proteomic data provide convergent evidence that decreased TCR diversity co‐occurs with elevated cytotoxic effector programs.

To further assess aging‐related effects on V(D)J rearrangement in T cells, we examined TCR variable region gene usage across the three age groups. The analysis revealed V and J gene usage frequencies changed with age, suggesting distinct clonal V(D)J rearrangements during aging. High‐frequency VJ combinations differed by age group: V12‐3/J2‐1 predominated in the young group, with a frequency of 0.218 among the top 20 combinations (Figure S8A); V5‐1/J1‐1 predominated in the middle‐aged group, with a frequency of 0.219 (Figure S8B); and V4‐1/J1‐5 predominated in the elderly group, with a frequency of 0.230 (Figure S8C). Circos plots of VJ combinations showed increasing clonotypic sites with age, suggesting increased antigen exposure during aging. Overall, these data indicate that aging immune cells exhibit increased clonality, reduced diversity, and altered TCR variable region gene usage, contributing to age‐related TCR repertoire abnormalities.

## 3 Discussion

Currently, several studies have examined age‐related alterations in the peripheral immune system. For example, the 10,000 Immunomes Project used microarray data to assess aging‐associated RNA expression changes in peripheral blood immune cells [28], and other studies have applied single‐cell RNA analysis to aging human peripheral blood [9, 21, 29]. However, these studies were largely limited to RNA‐level analyses, with limited attention to proteomics. Moreover, most used single‐omics approaches, leaving the multi‐omics interactions underlying immune aging incompletely characterized.

Here, we comprehensively investigated aging‐associated changes in peripheral immune cells using multi‐omics sequencing. Normal immune function requires balanced immune cell abundance, function, TCR repertoire diversity, and proportions of undifferentiated cells and clonally expanded lymphocytes, enabling both broad responses to unknown antigens and specific responses to antigenic challenges. Aging disrupts this balance. For example, adult thymic atrophy reduces T lymphocyte output; in young adults, less than 20% of new T cells are thymus derived, declining to less than 1% after age 50 [30]. Peripheral homeostatic proliferation compensates for reduced T‐cell output [31], but our study showed marked age‐associated declines in naïve CD4 and CD8 T lymphocyte proportions, accompanied by reduced absolute lymphocyte counts. This indicates that peripheral homeostatic proliferation cannot fully compensate for the reduced T‐cell output caused by thymic atrophy as aging progresses. The diminished effectiveness of homeostatic proliferation may be attributed to structural changes, such as tissue fibrosis, in the peripheral secondary lymphoid organs with increasing age, leading to functional degradation. This fibrosis may also be related to the weakened vaccine response observed in the aging population [32]. Neutrophils play a crucial role in inflammatory responses, and the increased proportion of neutrophils during the aging process suggests a gradual emergence of an immunoinflammatory state. Recent studies have indicated that neutrophils can induce adjacent cell telomere dysfunction in a ROS‐dependent manner, thereby triggering cellular senescence [33].

At the transcriptomic level, our analyses revealed that aging was associated with enhanced granulocyte activation and inflammation‐related immune responses, consistent with an immunoinflammatory state and previous reports [2, 34]. Integrative lncRNA–mRNA analysis suggested that age‐upregulated lncRNAs contribute to this immunoinflammatory state by modulating mRNA expression, whereas age‐downregulated lncRNAs mediate RNA splicing dysregulation through splicing factors. Bulk RNA‐seq, single‐cell RNA‐seq, and proteomics consistently identified substantial RNA splicing dysregulation in peripheral blood leukocytes from elderly individuals, mainly due to downregulation of splicing factors. RNA splicing is a fundamental post‐transcriptional regulatory mechanism that generates protein diversity in eukaryotes [35, 36], and its dysregulation may play a key role in immunosenescence. In this study, we identified previously known aging‐inducing genes such as AKT1, IRF5, and SREBF1, as well as aging‐inhibiting genes such as FOS, NEK1, RUVBL2, and SGK1, exhibiting RNA splicing alterations during aging. While this serves as a clue, elucidating whether the splicing of these genes indeed exerts a significant impact on the process of immune aging necessitates labor‐intensive experiments to systematically investigate the precise function of these splicing events.

Furthermore, integrative analysis of RNA splicing and protein profiles showed that age‐related splicing changes mainly affect genes involved in protein translation, processing, and degradation, thereby reshaping the protein landscape of aging leukocytes. Proteomics revealed age‐associated changes in multiple aging‐regulatory proteins, reflecting an overall aging trend in peripheral immune cells, and protein profile alterations also indicated T‐cell functional decline. These findings highlight RNA splicing as a key contributor to immune aging through modulation of protein profiles. In addition, integrative lncRNA–protein analysis showed that age‐associated lncRNAs primarily mediate protein downregulation, with downregulated proteins mainly enriched in RNA splicing. Together, these results suggest that lncRNAs regulate mRNAs and proteins involved in RNA splicing, thereby affecting downstream splicing events and disrupting protein translation, modification, and degradation. This multi‐molecular interaction network may substantially contribute to immunosenescence.

Despite the integrative multi‐omics design and the consistency of findings across bulk RNA‐seq, single‐cell RNA‐seq, TCR‐seq, and proteomics, several limitations should be noted when interpreting our results. First, our ability to link alternative splicing to proteoform‐level consequences is constrained by the depth and design of standard discovery proteomics. After re‐searching the MS data against an isoform‐aware ENSEMBL database, we identified 20,705 protein entries and quantified 4111 isoforms. However, only 224 genes had >1 quantified isoform and only 12 transcript–protein isoform pairs showed positive association. This low isoform resolution reflects the well‐known limitation that many transcript isoforms do not produce isoform‐unique tryptic peptides or yield peptides that are too short/low abundance to be detected. Besides, although aging‐associated splicing may generate novel antigenic peptides, our current cohort size and bulk proteomic design lack the sensitivity and scale required for robust neoantigen discovery. Confident identification of low‐abundance, sample‐specific splicing‐derived neo‐peptides typically requires substantially larger cohorts and dedicated immunopeptidomic or targeted mass‐spectrometry approaches. Future studies combining large, deeply sequenced cohorts with immunopeptidomics and targeted validation will be needed to directly identify neoantigenic peptides generated by aging‐associated splicing events.

In summary, our study represents the first comprehensive integration of multi‐omics sequencing data from the peripheral blood leukocytes of the same aging cohort. We intricately analyzed molecular alterations within peripheral blood leukocytes during aging, elucidating the interconnections among these molecules and their impact on immune system function. This work provides a global omics resource for understanding immune aging. Subsequent investigations may focus on associating the molecular changes in specific cell types with the physiological phenotypes of aging and unraveling the causal relationships among diverse molecular changes.

## 4 Materials and Methods

### 4.1 Study Participants and Sample Collection

All subjects provided written informed consent before sample collection. The study was conducted in accordance with the Declaration of Helsinki and was approved by the Ethics Committee of the Cancer Hospital, Chinese Academy of Medical Sciences. Sixty‐nine healthy Chinese subjects aged 23–75 years were enrolled in this study (Table S1). Peripheral venous blood samples were collected from each participant. Subjects with autoimmune diseases or other conditions affecting immune function, such as diabetes and thyroid disorders, were excluded. All enrolled participants had normal blood routine indicators and normal tumor marker levels. Peripheral blood leukocytes were isolated for RNA sequencing, TCR repertoire sequencing, and proteomic analyses. Detailed sample processing procedures are provided in the Supporting Information Materials and Methods.

### 4.2 Quality Control and Processing of RNA Sequencing Data

Raw RNA sequencing data were evaluated using FastQC, and adapters and low‐quality reads were removed using Trim‐Galore. ASEs were identified and quantified using SpliceSeq [37]. Gene and transcript expression levels were calculated using Salmon [38].

### 4.3 TCR Repertoire Sequencing Data Processing

Each TCR sequencing sample generated 9GB raw data. TCR repertoire sequencing data were processed based on unique molecular identifiers (UMIs) using MIGEC software (https://github.com/mikessh/migec/releases; Moscow, Russia). CDR3 sequences and V, D, and J genes were aligned using MiXCR (https://github.com/milaboratory/mixcr; Moscow, Russia). Clonotypes were analyzed using the R package “tcR” [39]. TCRB clonal diversity was assessed by observed clonotypes and diversity indices, including Efron–Thisted [34] and Chao1 [40]. VDJtools was used to calculate diversity metrics across age groups, with higher values indicating greater diversity.

### 4.4 Immune Cell Abundance Estimation and mRNA Clustering

Immune cell abundance in peripheral blood was estimated from transcriptomic data using CIBERSORT with LM22 reference genes [41], and immune cell signatures were validated using TIMER2.0 [42]. Spearman correlation analysis was performed between mRNA expression and age. mRNAs with *p* < 0.05 and correlation coefficient > 0.25 were retained. ClusterGVis (https://github.com/junjunlab/ClusterGVis) was used to identify temporal expression patterns across the three age groups. Only mRNAs with membership ≥ 0.4 in each subcluster were retained. The representative function of each cluster was determined by GO enrichment analysis.

### 4.5 Transcription Factor Regulation Enrichment and Activity Inference

DoRothEA human regulons with confidence levels A/B/C were used to test whether TF targets were enriched among age‐associated mRNAs. For each TF, a right‐tailed hypergeometric/Fisher's exact test was performed, and *p*‐values were adjusted using the Benjamini–Hochberg method. TFs with BH‐adjusted *p* < 0.05 were considered significantly enriched.

Sample‐level TF activities were inferred using the VIPER algorithm based on DoRothEA regulons and the normalized, batch‐corrected expression matrix. The resulting TF activity matrix was used for downstream correlation analyses and visualization. Detailed information is provided in the Supporting Information Materials and Methods.

### 4.6 Promoter Motif Scanning and Enrichment

Promoter regions of age‐associated genes were scanned for TF binding motifs to support direct DNA binding. Promoters were defined as −2000 to +200 bp relative to the transcription start site. Position weight matrices were obtained from JASPAR2022(PFMatrixList). Motif enrichment was tested using Fisher's exact test, followed by Benjamini–Hochberg correction. Motifs with BH‐adjusted *p* < 0.05 were considered significantly enriched. Detailed procedures are provided in the Supporting Information Materials and Methods.

### 4.7 Identification and Functional Analysis of Age‐Associated lncRNAs

Spearman correlation analysis was performed between lncRNA expression and age. lncRNAs with adjusted *p* < 0.05 were retained as age‐associated lncRNAs. Co‐expression analysis was then performed between lncRNAs and mRNAs, and pairs with correlation coefficient > 0.85 were considered co‐expressed. Functional enrichment analysis was performed using the LncSEA database [24], and subcellular localization was predicted using iLoc‐lncRNA [43].

### 4.8 Processing and Analysis of Single‐Cell Transcriptome Data

Single‐cell RNA sequencing data of aging‐associated human peripheral immune cells were obtained from the Genome Sequence Archive (https://bigd.big.ac.cn/gsa‐human/) under Project Accession No. PRJCA002865 and GSA Accession No. HSA000203. The dataset included peripheral blood mononuclear cells from 16 healthy subjects. Seurat was used for quality control and downstream analysis [44]. Cells with > 200 genes/cell, < 2500 genes/cell, and < 20% mitochondrial gene content were retained. After normalization, principal component analysis, clustering, and UMAP visualization were performed. Cell clusters were annotated using canonical immune cell markers. Differentially expressed genes and conserved marker genes were identified using “FindMarkers” and “FindConservedMarkers,” respectively. Detailed information is provided in the Supporting Information Materials and Methods.

### 4.9 Proteomics Sample Selection and Quality Control

Of the 69 participants, 48 samples passed predefined post‐extraction quality control (QC) criteria and were used for LC‐MS/MS analysis. QC metrics included total protein yield, protein integrity, contamination or degradation assessment, and peptide yield after digestion. Samples that failed QC (*n* = 21) were excluded. All samples were processed using the same protocol and reagents in randomized batches to reduce systematic bias. The 48 QC‐passed samples were subsequently processed for LC‐MS/MS and downstream analysis.

### 4.10 RNA Splicing Analysis

ASEs detected in at least two samples within each age group were retained. Each ASE was quantified using a percent spliced‐in (PSI) value. Spearman correlation analysis was performed between PSI values and age. *p*‐values were adjusted using the Benjamini–Hochberg method, and ASEs with adjusted *p* < 0.05 were considered age associated. GO enrichment analysis of genes related to age‐associated ASEs was performed using “ClusterProfilers” [45]. Pfam [46], CPC2 [47], SignalIP [48], NetSurfP‐2.0 [49], and TappAS [50] were used to investigate functional differences among isoforms.

### 4.11 Flow Cytometry Analysis

To validate the RNA sequencing findings, peripheral blood samples were collected from an independent cohort of 58 healthy subjects aged 21–65 years. Peripheral blood mononuclear cells were isolated and analyzed by flow cytometry. Naïve CD4 T cells were defined as CD3+CD4+CD45RA+ cells. Compensation and gating strategies were established using fluorescence‐minus‐one controls. Data were analyzed using FlowJo, and the proportion of naïve CD4 T cells was calculated relative to total CD4+ T cells. Detailed staining procedures and antibody information are provided in the Supporting Information Materials and Methods.

## Author Contributions

Ting Xiao, Shujun Cheng, and Lin Feng were the overall principal investigators who conceived the study design, obtained financial support, supervised the entire study, and reviewed the manuscript. Ting Xiao also contributed to manuscript revision. Quanyou Wu was responsible for the study design, performed the data analyses, interpreted the results, wrote the initial manuscript, and contributed to manuscript revision. Botao Zhang, Xiaochen Zhi, and Qi Zhang performed the data analyses and sample collection. Xiaochen Zhi also contributed to manuscript revision and prepared the graphical abstract. Kai Zhang supervised the sample collection and was responsible for information curation. Kai Zhang and Yaru Wang oversaw statistical analyses and interpreted the results. Kaitai Zhang reviewed the manuscript. All authors have read and approved the article.

## Funding

This work was supported by the National Natural Science Foundation of China (Grant No. 82273120), CAMS Innovation Fund for Medical Sciences (CIFMS) (Grant No. 2021‐I2M‐1‐50, 2023‐I2M‐2‐004), and the Postdoctoral Fellowship Program of CPSF under Grant Number GZC20231822. Funders only provided funding and had no role in the study design, data collection, data analysis, interpretation, and writing of the report.

## Ethics Statement

This study was approved by the Ethics Committee of National Cancer Center/Cancer Hospital, Chinese Academy of Medical Sciences and Peking Union Medical College (project number 23/240‐3982). All subjects involved in this study provided written informed consent before sample collection.

## Conflicts of Interest

The authors declare no conflicts of interest.

## Supporting information




**Figure. S1** | PCA of normalized transcriptomic profiles with samples colored by collection year (A) and by chronological age (continuous gradient) (B). Each point represents one sample.
**Figure. S2** | (A) Clustering of peripheral blood leukocyte from two age groups, predominantly clustering into five major cell types: T cells (TC), natural killer cells (NK), B cells (BC), monocytes (MC), and dendritic cells (DC). YS: Young scRNA‐seq group; AS: Aging scRNA‐seq group. (B) T cell clustering analysis. (C) CD4 T cell and CD8 T cell clustering analysis. (D) NK cell clustering analysis. (E) B cell clustering analysis.
**Figure. S3** | Immunocytes in the aging cohort exhibit distinctive age‐associated molecular expression patterns.
**Figure. S4** | Differential analysis of transcriptomes and GO enrichment analysis for various immune cell subtypes. (A‐B) Upregulated and downregulated genes along with their biologically enriched functions in different CD4 T cell subtypes during aging. (C‐D) Upregulated and downregulated genes along with their biologically enriched functions in different NK cell subtypes during aging. (E‐F) Upregulated and downregulated genes along with their biologically enriched functions in different B cell subtypes during aging. Note: Red and blue squares represent significantly upregulated and downregulated biological processes, respectively; gray squares represent processes that are not significantly enriched. The top 10 significantly enriched biological processes are visualized for each analysis.
**Figure. S5** | (A) Number of identified proteins and peptides in each sample across the three age groups. (B‐C) Boxplots illustrating the distribution of raw protein quantification values and normalized protein quantification values for each sample. (D) GO Biological Process enrichment of 130 proteins identified exclusively in the elderly group. Dot size represents the number of genes mapped to each term; dot color indicates −log10(p‐value). Terms are ordered by gene ratio (largest at top). (E) Volcano plot of per‐gene mRNA–protein concordance. Each point represents one gene; the x‐axis shows the Spearman correlation coefficient between mRNA and protein abundance and the y‐axis shows the corresponding significance. Genes are categorized by correlation strength using absolute r: weak (0.1 ≤ |r| < 0.3), moderate (0.3 ≤ |r| < 0.5) and strong (|r| ≥ 0.5).
**Figure. S6** | The association between the expression of age‐associated lncRNAs and age‐associated proteins. The orange and blue circles represent lncRNAs that are upregulated and downregulated with age, respectively. Similarly, the purple and green circles represent proteins that are upregulated and downregulated with age, respectively. The pink and green edges indicate positive and negative correlations, respectively. Splicing‐related proteins are labeled in blue within the green circles, while proteins known to inhibit cellular senescence are labeled in red.
**Figure. S7** | Transcript–protein correlations for 12 isoforms supported by isoform‐unique peptides. Scatter plots show protein isoform intensities (log2‐transformed, sample median‐centered) versus transcript abundances (log2(TPM+1)) across 48 paired samples. Spearman correlation coefficient and nominal p‐value are indicated in each panel.
**Figure. S8** | Circos plot illustrating the differences in the frequency of V and J gene usage among the three age groups. The width of the outer circular sectors represents the relative frequency of V or J genes, while the width of the connections between V and J gene combinations indicates their frequency of usage in specific age groups.

**Table S1**. Information of each subject in the aging cohort.
**Table S2**. Information of each subject in the flow cytometry cohort.
**Table S3**. Age‐associated lncRNAs.
**Table S4**. Molecular markers for 18 subtypes of immune cells.
**Table S5**. Age‐associated proteins.
**Table S6**. Correlation between age‐associated lncRNAs and age‐associated proteins.
**Table S7**. Age‐associated ASEs.
**Table S8**. Correlation between ASEs of genes involved in protein regulation and the expression of protein profiles.
**Table S9**. Sequences of primers used in TCRB library construction.

## Data Availability

The raw RNA‐seq and TCR sequencing data generated in this study have been deposited in the Genome Sequence Archive (GSA‐Human) at the National Genomics Data Center, China National Center for Bioinformation/Beijing Institute of Genomics, Chinese Academy of Sciences [51, 52] under accession numbers HRA016187 and HRA015888, and are publicly accessible at https://ngdc.cncb.ac.cn/gsa‐human. The raw mass‐spectrometry (proteomics) data have been deposited in the iProX repository under accession number IPX0015369000 and are publicly accessible at https://www.iprox.cn/. All analysis code used in this study is available at https://github.com/s01edAd/AgingCohort.

## References

[1] P. P. Singh , B. A. Demmitt , R. D. Nath , and A. Brunet , “The Genetics of Aging: A Vertebrate Perspective,” Cell 177 (2019): 200–220. 30901541 10.1016/j.cell.2019.02.038 PMC7592626

[2] C. López‐Otín , M. A. Blasco , L. Partridge , M. Serrano , and G. Kroemer , “Hallmarks of Aging: An Expanding Universe,” Cell 186 (2023): 243–278. 36599349 10.1016/j.cell.2022.11.001

[3] N. P. Weng , “Aging of the Immune System: How Much Can the Adaptive Immune System Adapt?,” Immunity 24 (2006): 495–499. 16713964 10.1016/j.immuni.2006.05.001 PMC2266981

[4] J. Nikolich‐Žugich , “The Twilight of Immunity: Emerging Concepts in Aging of the Immune System,” Nature Immunology 19 (2018): 10–19. 29242543 10.1038/s41590-017-0006-x

[5] C. Franceschi , P. Garagnani , P. Parini , C. Giuliani , and A. Santoro , “Inflammaging: A New Immune‐Metabolic Viewpoint for Age‐Related Diseases,” Nature Reviews Endocrinology 14 (2018): 576–590. 10.1038/s41574-018-0059-4 30046148

[6] D. A. Mogilenko , O. Shpynov , P. S. Andhey , et al., “Comprehensive Profiling of an Aging Immune System Reveals Clonal GZMK(+) CD8(+) T Cells as Conserved Hallmark of Inflammaging,” Immunity 54 (2021): 99–115.e12. 33271118 10.1016/j.immuni.2020.11.005

[7] M. J. Peters , R. Joehanes , L. C. Pilling , et al., “The Transcriptional Landscape of Age in Human Peripheral Blood,” Nature Communications 6 (2015): 8570. 10.1038/ncomms9570 PMC4639797 26490707

[8] L. M. Reynolds , J. Ding , J. R. Taylor , et al., “Transcriptomic Profiles of Aging in Purified Human Immune Cells,” BMC Genomics 16 (2015): 333. 25898983 10.1186/s12864-015-1522-4 PMC4417516

[9] O. J. Luo , W. Lei , G. Zhu , et al., “Multidimensional Single‐Cell Analysis of Human Peripheral Blood Reveals Characteristic Features of the Immune System Landscape in Aging and Frailty,” Nature Aging 2 (2022): 348–364. 37117750 10.1038/s43587-022-00198-9

[10] A. Ghazalpour , B. Bennett , V. A. Petyuk , et al., “Comparative Analysis of Proteome and Transcriptome Variation in Mouse,” Plos Genetics 7 (2011): e1001393. 21695224 10.1371/journal.pgen.1001393 PMC3111477

[11] J. Bathke , A. Konzer , B. Remes , M. McIntosh , and G. Klug , “Comparative Analyses of the Variation of the Transcriptome and Proteome of Rhodobacter Sphaeroides Throughout Growth,” BMC Genomics 20 (2019): 358. 31072330 10.1186/s12864-019-5749-3 PMC6509803

[12] H. S. Li , C. M. Liu , S. F. Zheng , et al., “RANKL/PD‐1 Dual Blockade Demonstrates Survival Benefit for Patients With Advanced Lung Adenocarcinoma Harboring KRAS Mutations,” Cell Reports Medicine 6 (2025): 102235. 40669444 10.1016/j.xcrm.2025.102235 PMC12281428

[13] H. S. Oh , Y. Le Guen , and N. Rappoport , “Plasma Proteomics Links Brain and Immune System Aging With Healthspan and Longevity,” Nature Medicine 31 (2025): 2703–2711. 10.1038/s41591-025-03798-1 PMC12353788 40634782

[14] D. A. Mogilenko , I. Shchukina , and M. N. Artyomov , “Immune Ageing at Single‐Cell Resolution,” Nature Reviews Immunology 22 (2022): 484–498. 10.1038/s41577-021-00646-4 PMC8609266 34815556

[15] O. I. Kiseleva , V. A. Arzumanian , Y. A. Ikhalaynen , I. Y. Kurbatov , P. A. Kryukova , and E. V. Poverennaya , “Multiomics of Aging and Aging‐Related Diseases,” International Journal of Molecular Sciences 25, no. 24 (2024): 13671. 39769433 10.3390/ijms252413671 PMC11677528

[16] Q. Gong , M. Sharma , M. C. Glass , et al., “Multi‐Omic Profiling Reveals Age‐Related Immune Dynamics in Healthy Adults,” Nature 648 (2025): 696–706. 41162704 10.1038/s41586-025-09686-5 PMC12711581

[17] M. Brazhnikov , T. Kusainova , A. S. Kopeykina , and I. A. Tarasova , “TMTCrunch: A Proteomic Atlas of Alternative Splicing for Predicting Splicing‐Induced Implications in Aging and Alzheimer's Disease,” Journal of Proteome Research 24 (2025): 5548–5563. 41024644 10.1021/acs.jproteome.5c00426

[18] S. Li , H. Lv , R. Zhang , et al., “Aging‐Related Alternative Splicing Drive Neoantigen Emergence Revealed by Transcriptome Analysis of 1,255 Human Blood Samples,” Frontiers in Aging 6 (2025): 1575862. 40417629 10.3389/fragi.2025.1575862 PMC12098113

[19] C. Buccitelli and M. Selbach , “mRNAs, Proteins and the Emerging Principles of Gene Expression Control,” Nature Reviews Genetics 21 (2020): 630–644. 10.1038/s41576-020-0258-4 32709985

[20] Z. Thomson , Z. He , E. Swanson , et al., “Trimodal Single‐Cell Profiling Reveals a Novel Pediatric CD8αα(+) T Cell Subset and Broad Age‐Related Molecular Reprogramming Across the T Cell Compartment,” Nature Immunology 24 (2023): 1947–1959. 37845489 10.1038/s41590-023-01641-8 PMC10602854

[21] M. Terekhova , A. Swain , P. Bohacova , et al., “Single‐Cell Atlas of Healthy Human Blood Unveils Age‐Related Loss of NKG2C(+)GZMB(‐)CD8(+) Memory T Cells and Accumulation of Type 2 Memory T Cells,” Immunity 56 (2023): 2836–2854.e9. 37963457 10.1016/j.immuni.2023.10.013

[22] B. Hu , R. R. Jadhav , and C. E. Gustafson , “Distinct Age‐Related Epigenetic Signatures in CD4 and CD8 T Cells,” Frontiers in Immunology 11 (2020): 585168. 33262764 10.3389/fimmu.2020.585168 PMC7686576

[23] R. Wang , C. P. Dillon , L. Z. Shi , et al., “The Transcription Factor Myc Controls Metabolic Reprogramming Upon T Lymphocyte Activation,” Immunity 35 (2011): 871–882. 22195744 10.1016/j.immuni.2011.09.021 PMC3248798

[24] J. Chen , J. Zhang , Y. Gao , et al., “LncSEA: A Platform for Long Non‐Coding RNA Related Sets and Enrichment Analysis,” Nucleic Acids Research 49 (2021): D969–D980. 33045741 10.1093/nar/gkaa806 PMC7778898

[25] X. Wang , S. G. Codreanu , B. Wen , et al., “Detection of Proteome Diversity Resulted From Alternative Splicing Is Limited by Trypsin Cleavage Specificity,” Molecular & Cellular Proteomics 17 (2018): 422–430. 29222161 10.1074/mcp.RA117.000155 PMC5836368

[26] P. Sinitcyn , A. L. Richards , R. J. Weatheritt , et al., “Global Detection of Human Variants and Isoforms by Deep Proteome Sequencing,” Nature Biotechnology 41 (2023): 1776–1786. 10.1038/s41587-023-01714-x PMC10713452 36959352

[27] J. P. Chou and R. B. Effros , “T Cell Replicative Senescence in Human Aging,” Current Pharmaceutical Design 19 (2013): 1680–1698. 23061726 10.2174/138161213805219711 PMC3749774

[28] K. A. Zalocusky , M. J. Kan , Z. Hu , et al., “The 10,000 Immunomes Project: Building a Resource for Human Immunology,” Cell Reports 25 (2018): 513–522.e3. 30304689 10.1016/j.celrep.2018.09.021 PMC6263160

[29] T. T. Karagiannis , T. W. Dowrey , C. Villacorta‐Martin , et al., “Multi‐Modal Profiling of Peripheral Blood Cells Across the Human Lifespan Reveals Distinct Immune Cell Signatures of Aging and Longevity,” EBioMedicine 90 (2023): 104514. 37005201 10.1016/j.ebiom.2023.104514 PMC10114155

[30] I. Bains , R. Antia , R. Callard , and A. J. Yates , “Quantifying the Development of the Peripheral Naive CD4+ T‐Cell Pool in Humans,” Blood 113 (2009): 5480–5487. 19179300 10.1182/blood-2008-10-184184 PMC2689049

[31] S. C. Jameson , “Maintaining the Norm: T‐Cell Homeostasis,” Nature Reviews Immunology 2 (2002): 547–556. 10.1038/nri853 12154374

[32] C. Kityo , K. N. Makamdop , M. Rothenberger , et al., “Lymphoid Tissue Fibrosis Is Associated With Impaired Vaccine Responses,” Journal of Clinical Investigation 128 (2018): 2763–2773. 29781814 10.1172/JCI97377 PMC6025977

[33] A. Lagnado , J. Leslie , M. H. Ruchaud‐Sparagano , et al., “Neutrophils Induce Paracrine Telomere Dysfunction and Senescence in ROS‐Dependent Manner,” Embo Journal 40 (2021): e106048. 33764576 10.15252/embj.2020106048 PMC8090854

[34] X. Li , C. Li , W. Zhang , Y. Wang , P. Qian , and H. Huang , “Inflammation and Aging: Signaling Pathways and Intervention Therapies,” Signal Transduction and Targeted Therapy 8 (2023): 239. 37291105 10.1038/s41392-023-01502-8 PMC10248351

[35] E. T. Wang , R. Sandberg , S. Luo , et al., “Alternative Isoform Regulation in Human Tissue Transcriptomes,” Nature 456 (2008): 470–476. 18978772 10.1038/nature07509 PMC2593745

[36] Q. Pan , O. Shai , L. J. Lee , J. Frey , and B. J. Blencowe , “Deep Surveying of Alternative Splicing Complexity in the Human Transcriptome by High‐Throughput Sequencing,” Nature Genetics 40 (2008): 1413–1415. 18978789 10.1038/ng.259

[37] M. C. Ryan , J. Cleland , R. Kim , W. C. Wong , and J. N. Weinstein , “SpliceSeq: A Resource for Analysis and Visualization of RNA‐Seq Data on Alternative Splicing and Its Functional Impacts,” Bioinformatics 28 (2012): 2385–2387. 22820202 10.1093/bioinformatics/bts452 PMC3436850

[38] R. Patro , G. Duggal , M. I. Love , R. A. Irizarry , and C. Kingsford , “Salmon Provides Fast and Bias‐Aware Quantification of Transcript Expression,” Nature Methods 14 (2017): 417–419. 28263959 10.1038/nmeth.4197 PMC5600148

[39] V. I. Nazarov , M. V. Pogorelyy , E. A. Komech , et al., “tcR: An R Package for T Cell Receptor Repertoire Advanced Data Analysis,” BMC Bioinformatics 16, no. 1 (2015): 175. 26017500 10.1186/s12859-015-0613-1 PMC4445501

[40] J. B. Hughes , J. J. Hellmann , T. H. Ricketts , and B. J. M. Bohannan , “Counting the Uncountable: Statistical Approaches to Estimating Microbial Diversity,” Applied and Environmental Microbiology 67 (2001): 4399–4406. 11571135 10.1128/AEM.67.10.4399-4406.2001 PMC93182

[41] A. M. Newman , C. L. Liu , M. R. Green , et al., “Robust Enumeration of Cell Subsets From Tissue Expression Profiles,” Nature Methods 12 (2015): 453–457. 25822800 10.1038/nmeth.3337 PMC4739640

[42] T. Li , J. Fu , Z. Zeng , et al., “TIMER2.0 for Analysis of Tumor‐Infiltrating Immune Cells,” Nucleic Acids Research 48 (2020): W509–W514. 32442275 10.1093/nar/gkaa407 PMC7319575

[43] Z. D. Su , Y. Huang , Z. Y. Zhang , et al., “iLoc‐lncRNA: Predict the Subcellular Location of lncRNAs by Incorporating Octamer Composition Into General PseKNC,” Bioinformatics 34 (2018): 4196–4204. 29931187 10.1093/bioinformatics/bty508

[44] A. Butler , P. Hoffman , P. Smibert , E. Papalexi , and R. Satija , “Integrating Single‐Cell Transcriptomic Data Across Different Conditions, Technologies, and Species,” Nature Biotechnology 36 (2018): 411–420. 10.1038/nbt.4096 PMC6700744 29608179

[45] G. Yu , L.‐G. Wang , Y. Han , and Q.‐Y. He , “clusterProfiler: An R Package for Comparing Biological Themes Among Gene Clusters,” Omics‐a Journal of Integrative Biology 16 (2012): 284–287. 22455463 10.1089/omi.2011.0118 PMC3339379

[46] M. Punta , P. C. Coggill , R. Y. Eberhardt , et al., “The Pfam Protein Families Database,” Nucleic Acids Research 40 (2011): D290–D301. 22127870 10.1093/nar/gkr1065 PMC3245129

[47] Y.‐J. Kang , D.‐C. Yang , L. Kong , et al., “CPC2: A Fast and Accurate Coding Potential Calculator Based on Sequence Intrinsic Features,” Nucleic Acids Research 45 (2017): W12–W16. 28521017 10.1093/nar/gkx428 PMC5793834

[48] J. J. Almagro Armenteros , K. D. Tsirigos , and C. K. Sønderby , “SignalP 5.0 Improves Signal Peptide Predictions Using Deep Neural Networks,” Nature Biotechnology 37 (2019): 420–423. 10.1038/s41587-019-0036-z 30778233

[49] M. S. Klausen , M. C. Jespersen , and H. Nielsen , “NetSurfP‐2.0: Improved Prediction of Protein Structural Features by Integrated Deep Learning,” BioRxiv 87, no. 6 (2018): 520–527. 10.1002/prot.25674 30785653

[50] L. de la Fuente , Á. Arzalluz‐Luque , M. Tardáguila , et al., “tappAS: A Comprehensive Computational Framework for the Analysis of the Functional Impact of Differential Splicing,” BioRxiv 21, no. 1 (2019): 119. 10.1186/s13059-020-02028-w PMC7236505 32423416

[51] S. Zhang , X. Chen , and E. Jin , “The GSA Family in 2025: A Broadened Sharing Platform for Multi‐Omics and Multimodal Data,” Genomics, Proteomics & Bioinformatics 23, no. 4 (2025): qzaf072. 10.1093/gpbjnl/qzaf072 PMC12451262 40857552

[52] CNCB–NGDC Members and Partners , “Database Resources of the National Genomics Data Center, China National Center for Bioinformation in 2026,” Nucleic Acids Research 54 (2026): D28–D47. 41359036 10.1093/nar/gkaf1172 PMC12807729

