# Supplementary material for: Cross‐Level Regulatory Interactions Underlying Human Immune Aging
Source: MedComm (2020). 2026 Jun 15;7(7):e70817. doi: 10.1002/mco2.70817 (PMC13269837; doi:10.1002/mco2.70817)
Supplement: Supplementary file 1 — Figure. S1 | PCA of normalized transcriptomic profiles with samples colored by collection year (A) and by chronological age (continuous gradient) (B). Each point represents one sample. Figure. S2 | (A) Clustering of peripheral blood leukocyte from two age groups, predominantly clustering into five major cell types: T cells (TC), natural killer cells (NK), B cells (BC), monocytes (MC), and dendritic cells (DC). YS: Young scRNA‐seq group; AS: Aging scRNA‐seq group. (B) T cell clustering analysis. (C) CD4 T cell and CD8 T cell clustering analysis. (D) NK cell clustering analysis. (E) B cell clustering analysis. Figure. S3 | Immunocytes in the aging cohort exhibit distinctive age‐associated molecular expression patterns. Figure. S4 | Differential analysis of transcriptomes and GO enrichment analysis for various immune cell subtypes. (A‐B) Upregulated and downregulated genes along with their biologically enriched functions in different CD4 T cell subtypes during aging. (C‐D) Upregulated and downregulated genes along with their biologically enriched functions in different NK cell subtypes during aging. (E‐F) Upregulated and downregulated genes along with their biologically enriched functions in different B cell subtypes during aging. Note: Red and blue squares represent significantly upregulated and downregulated biological processes, respectively; gray squares represent processes that are not significantly enriched. The top 10 significantly enriched biological processes are visualized for each analysis. Figure. S5 | (A) Number of identified proteins and peptides in each sample across the three age groups. (B‐C) Boxplots illustrating the distribution of raw protein quantification values and normalized protein quantification values for each sample. (D) GO Biological Process enrichment of 130 proteins identified exclusively in the elderly group. Dot size represents the number of genes mapped to each term; dot color indicates −log10(p‐value). Terms are ordered by gene ratio [file MCO2-7-e70817-s002.docx]

**Supplementary Materials for**

Cross-level Regulatory Interactions Underlying Human Immune Aging

Quanyou Wu^1,3#^, Botao Zhang^1,2#^, Xiaochen Zhi^1,#^, Qi Zhang^1,#^, Kai Zhang^4^, Yaru Wang^1^, Kaitai Zhang^1^, Lin Feng^1,^*, Shujun Cheng^1,^*, Ting Xiao^1,^*

Correspondence to: [xiaot@cicams.ac.cn](mailto:xiaot@cicams.ac.cn); [chengshj@cae.cn](mailto:chengshj@cae.cn); [fenglin@cicams.ac.cn](mailto:fenglin@cicams.ac.cn)

**This PDF file includes:**

Materials and Methods

Figures. S1 to S8

Tables S1 to S9 (See attached Excel files)

**Materials and Methods**

**Sample collection and blood processing**

Samples from subjects aged 23–40 years were obtained in April 2017 from employees of the Cancer Hospital, Chinese Academy of Medical Sciences, totaling 17 cases. Samples from subjects aged 47–75 years were collected from individuals undergoing cancer screening at the Cancer Hospital Prevention and Examination Center from March 2017 to March 2019, totaling 50 cases. Six milliliters of peripheral whole blood were collected using EDTA-K2 anticoagulant vacuum tubes. After collection, samples were transported on ice, temporarily stored at 4°C and processed for white blood cell separation within 6 hours.

**Isolation of peripheral blood leukocytes and RNA extraction**

Peripheral whole blood was centrifuged for blood cell isolation. Red blood cells were lysed using erythrocyte lysis buffer (Qiagen, Germany), and leukocytes were obtained within 6 hours of collection. Total RNA was extracted using TRIzol solution (Invitrogen, USA). RNA purity was assessed using a NanoDrop2000 spectrophotometer (Thermo Fisher Scientific, USA), and samples with OD_260/280_ ≥ 1.9 were retained. RNA integrity was further evaluated using a Bioanalyzer 2100 (Agilent, USA). All RNA samples had RNA integrity number > 8.0.

**Construction of cDNA library and RNA sequencing**

A lncRNA sequencing strategy was used for transcriptomic profiling because it can capture both lncRNA and mRNA information. A total of 2 μg RNA per sample was used for library construction. The procedure involved the removal of ribosomal RNA (rRNA) using the Epicentre Ribo-zero^TM^ rRNA Removal Kit, followed by ethanol precipitation to eliminate residual free rRNA. Subsequently, the NEBNext Ultra^TM^ Directional RNA Library Prep Kit, compatible with the Illumina platform, was employed to construct sequencing libraries from rRNA-depleted RNA samples. The library construction process began with sample fragmentation in NEBNext First Strand Synthesis Reaction Buffer (5X) under high-temperature conditions. Subsequently, the first-strand cDNA was synthesized using random hexamers and M-MuLV reverse transcriptase (RNaseH^-^). The second-strand DNA was then synthesized using DNA polymerase I and RNase H, with dTTP replaced by dUTP in the reaction buffer. After adenylation of the 3' ends of DNA fragments, NEBNext sequencing adapters with hairpin structures were ligated for subsequent hybridization. To selectively purify cDNA fragments with lengths ranging from 250 to 300 bp, AMPure XP nucleic acid purification reagents were employed. The library fragments, subjected to 3μl USER Enzyme treatment, were reacted at 37°C for 15 minutes. Subsequent PCR was performed using Phusion high-fidelity DNA polymerase, universal PCR primers, and index (X) primers. The final products underwent purification (AMPure XP nucleic acid purification reagents), and the library quality was assessed on an Agilent Bioanalyzer 2100 system. Following library construction, sequencing was conducted on the Illumina NovaSeq 6000 platform using the PE150 strategy, generating a sequencing data volume of 12G raw data per sample. Both RNA library construction and sequencing were performed by NovoGen Inc.

**Construction and sequencing of TCR repertoire library**

For TCR library preparation, 700 ng total RNA was used per patient. TCRB-specific cDNA was synthesized using SuperScript II reverse transcriptase (Invitrogen; cat. no. 18064), a TCRB C-region primer and UMIs incorporated in TSOs. Redundant TSOs were removed using Uracil-DNA-Glycosylase (New England Biolabs; cat. no. M0280), and cDNA was amplified using three nested and step-out PCR assays with Q5 Hot-start High-Fidelity DNA Polymerase (New England Biolabs; cat. no. M0493). The initial nested PCR and the subsequent nested and step-out PCR were conducted as follows: 1 cycle at 94 °C for 90 s; 10 cycles of denaturation at 94 °C for 10 s, annealing at 63 °C for 20 s, and extension at 72 °C for 40 s; followed by a final extension cycle at 72 °C for 4 min. The third step-out PCR, introducing an additional semisequencing adaptor for clustering, proceeded with: 1 cycle at 94 °C for 90 s; 16 cycles of denaturation at 94 °C for 10 s, annealing at 60 °C for 20 s, and extension at 72 °C for 40 s; concluding with a final extension cycle at 72 °C for 4 min. Purification of PCR products from each round was performed using a QIAquick PCR Purification Kit (Qiagen; cat. no. 28106). These purified products were then fractionated to serve as templates for the subsequent PCR. Detailed primer sequences utilized for TCRB library preparation can be found in Table S9. Ultimately, eligible TCRB libraries underwent clustering and sequencing on an Illumina HiSeq XTen platform (150 bp paired-end reads, Illumina, USA).

**Detailed transcription factor activity inference**

DoRothEA regulons with confidence levels A/B/C were converted to VIPER regulon format. When explicit edge weights were unavailable, confidence-based weights were assigned as A = 1.0, B = 0.66 and C = 0.33. The mor column was used to define the mode of regulation as +1/−1. VIPER was run on the normalized, batch-corrected expression matrix with default settings unless otherwise indicated.

**Detailed promoter motif scanning and enrichment**

Promoter sequences were defined as −2000 to +200 bp relative to the transcription start site using hg38 coordinates. TSS locations were obtained from Ensembl via biomaRt and restricted to canonical chromosomes (chr1–22, chrX, chrY). Sequences were extracted from BSgenome.Hsapiens.UCSC.hg38. PWMs were obtained from JASPAR2022 (PFMatrixList). We scanned promoters with motifmatchr::matchMotifs() supported by the R package. For each motif, we computed a binary presence/absence matrix for enrichment analysis. All motif scanning used JASPAR2022 and hg38 PWM/sequence resources.

**Single-cell RNA-seq library preparation and analysis details**

For library preparation, single-cell RNA libraries of peripheral blood mononuclear cells from 16 healthy subjects were constructed using the Chromium Single Cell 5′ v2 Reagent from 10x Genomics. Subsequently, these libraries were subjected to high-throughput sequencing using the Illumina NovaSeq6000 platform. After quality control, data normalization and detection of 5,000 variable genes were performed for principal component analysis. The initial 30 PCA components were utilized to cluster diverse cell types at a resolution of 0.5. Visualization of distinct clusters was achieved using the UMAP method. Differential expression analysis and identification of marker genes were carried out using the "FindMarkers" function and the "FindConservedMarkers" function with default thresholds, respectively.

**DDA library building and LC-MS/MS settings**

DDA library building and following DIA analysis were performed with thermo Oribitrap Fusion Lumos coupled to an EASY-nano-LC 1200 system. The mobile phase consisted of 0.1% formic acid in water (A), and 0.1% formic acid/80% acetonitrile (B). For the DDA library building, the mobile phase flow rate was 600 nl/min and subjected into a gradient profile which was set as follows: 11% buffer B for 3 min, 14% buffer B for 39 min, 37% buffer B for 11 min, 95% buffer B for 1 min, 95% buffer B for 6 min. The Oribitrap Fusion Lumos mass spectrometer parameters were as follows: MS spectra were collected from 350 to 1550 m/z at a resolution of 120 K along with data-dependent Orbitrap HCD MS/MS spectra at a resolution of 15 K. Ions selected for MS/MS were fragmented using a normalized collision energy of 32%. Dynamic exclusion time was set to 18 s. Peptides were analyzed with First Mass 100 with 15,000 resolution and AGC target of 5e4.

**Mass spectrometry database search parameters**

Mass spectrometry files were analyzed using Proteome Discoverer (PD) v2.2 against the human UniProt SwissProt database (SwissProt202002, 20350 entries). For PD, the parameters used included trypsin as a digestive enzyme, two missing cleavage sites, Carbamidomethyl (C) as a fixed modification, and Oxidation (M) and Acetyl (protein N-terminus) as variable modifications. To ensure the accuracy of analysis, we removed proteins with missing expression values in more than 25% of participants. To encompass diverse functional immune cell subtypes, we curated a reference gene list by extracting characteristic molecules from 18 immune cell subtypes, as documented in Table S4, drawing from pertinent literature reports [1, 2]. Employing this reference gene list, we conducted immune scoring through single-sample gene set enrichment analysis. Genes related to cellular senescence were obtained from the CellAge database [3], while the gene set for TCR signaling pathway was sourced from the immPort database [4].

**Flow cytometry staining and gating procedures**

Peripheral blood mononuclear cells were isolated by density gradient centrifugation using Human Peripheral Blood Lymphocyte Separation Solution (LTS10771, TBD Science, Tianjin, China) and resuspended in Stain Buffer (FBS) (#554656, BD Biosciences). Cells were stained with anti-CD3 (UCHT1, BD Biosciences), anti-CD4 (RPA-T4, BD Biosciences) and anti-CD45RA (HI100, BD Biosciences). After staining, cells were washed, resuspended and analyzed using a BD LSR II flow cytometer. Naïve CD4 T cells were identified as CD3+CD4+CD45RA+ cells. Compensation and gating strategies were established using fluorescence-minus-one controls. Data were analyzed using FlowJo software (Version 10.8.1), and naïve CD4 T cell proportions were calculated relative to total CD4+ T cells.

**References**

1. Şenbabaoğlu Y, Gejman RS, Winer AG, Liu M, Van Allen EM, de Velasco G, et al. Erratum to: Tumor immune microenvironment characterization in clear cell renal cell carcinoma identifies prognostic and immunotherapeutically relevant messenger RNA signatures. Genome Biol. 2017; 18: 46.

2. Tamborero D, Rubio-Perez C, Muiños F, Sabarinathan R, Piulats JM, Muntasell A, et al. A Pan-cancer Landscape of Interactions between Solid Tumors and Infiltrating Immune Cell Populations. Clin Cancer Res. 2018; 24: 3717-28.

3. Avelar RA, Ortega JG, Tacutu R, Tyler EJ, Bennett D, Binetti P, et al. A multidimensional systems biology analysis of cellular senescence in aging and disease. Genome Biol. 2020; 21: 91.

4. Bhattacharya S, Dunn P, Thomas CG, Smith B, Schaefer H, Chen J, et al. ImmPort, toward repurposing of open access immunological assay data for translational and clinical research. Sci Data. 2018; 5: 180015.

**Supplementary Figure 1**


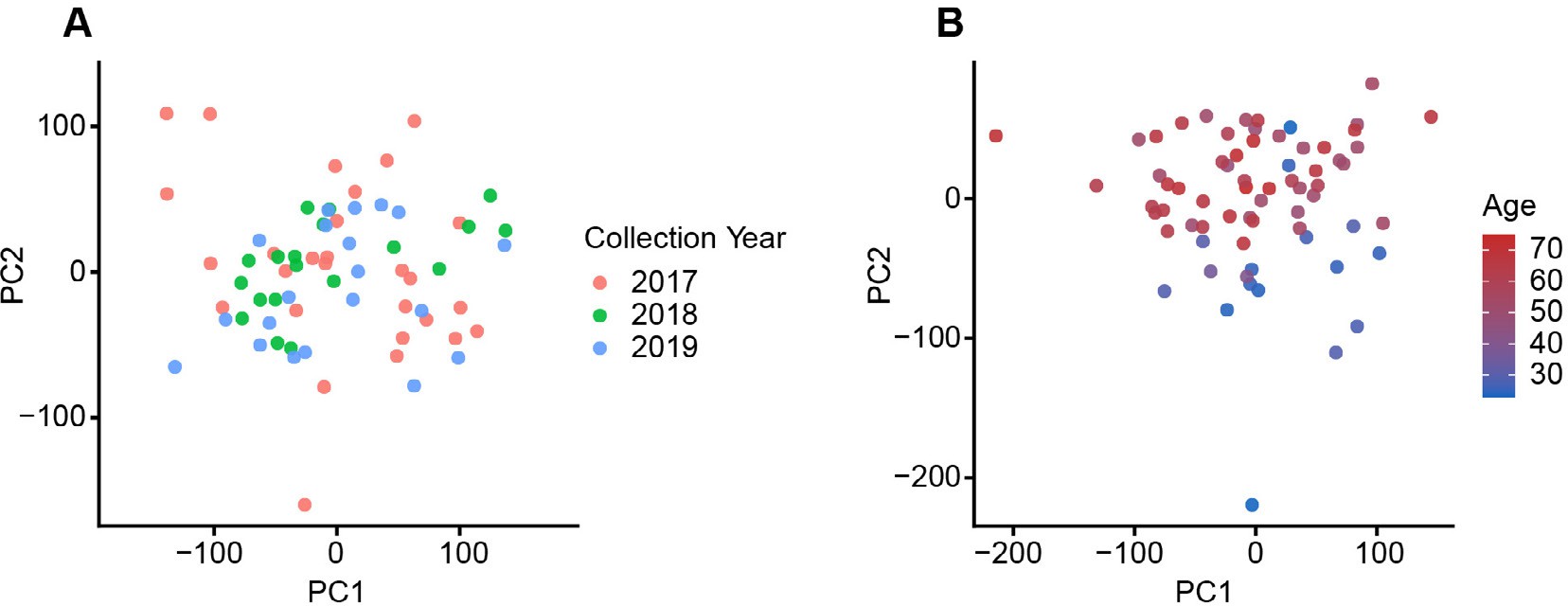


Fig. S1 | PCA of normalized transcriptomic profiles with samples colored by collection year (A) and by chronological age (continuous gradient) (B). Each point represents one sample.

**Supplementary Figure 2**


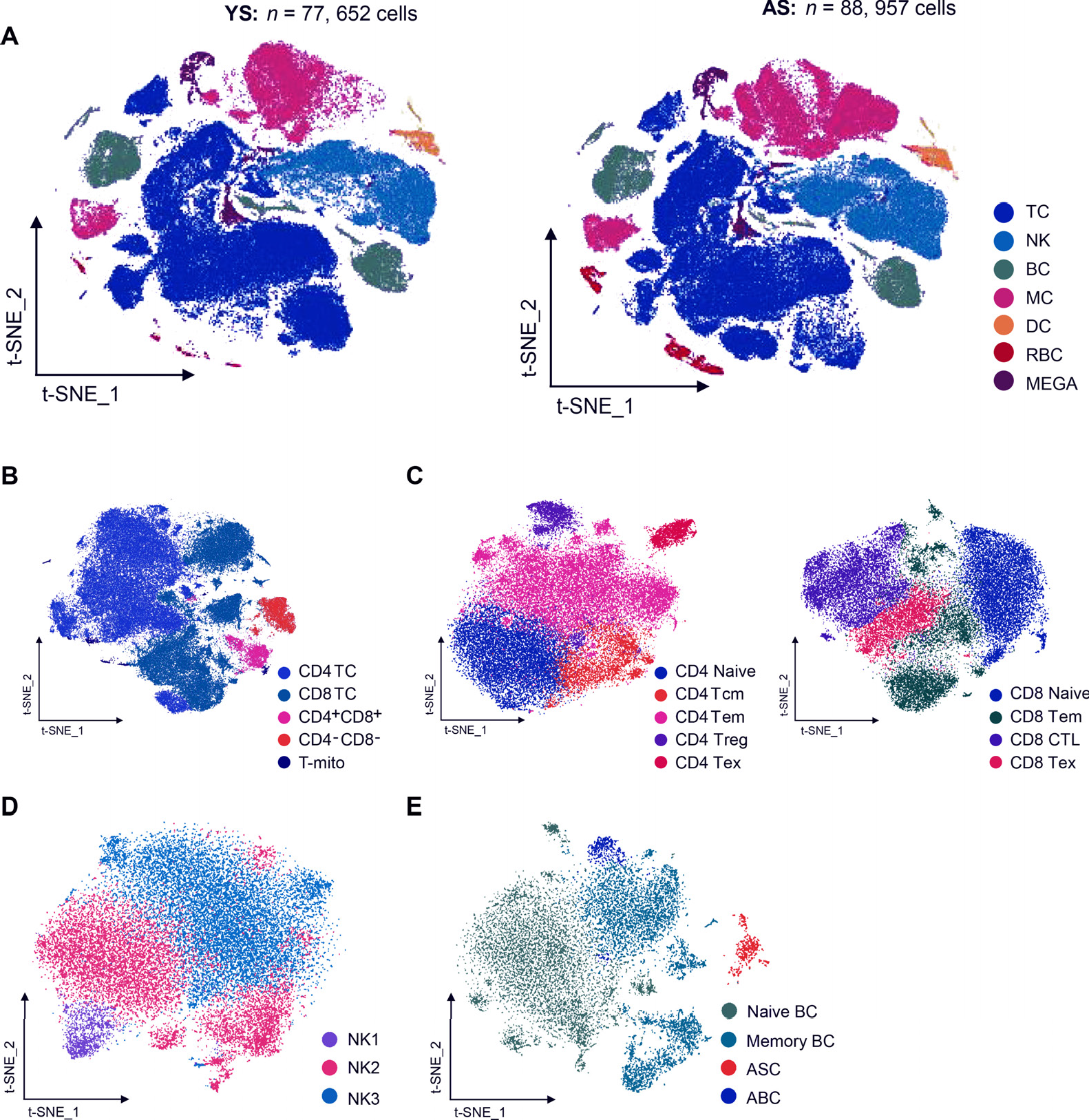


Fig. S2 | (A) Clustering of peripheral blood leukocyte from two age groups, predominantly clustering into five major cell types: T cells (TC), natural killer cells (NK), B cells (BC), monocytes (MC), and dendritic cells (DC). YS: Young scRNA-seq group; AS: Aging scRNA-seq group. (B) T cell clustering analysis. (C) CD4 T cell and CD8 T cell clustering analysis. (D) NK cell clustering analysis. (E) B cell clustering analysis.

**Supplementary Figure 3**


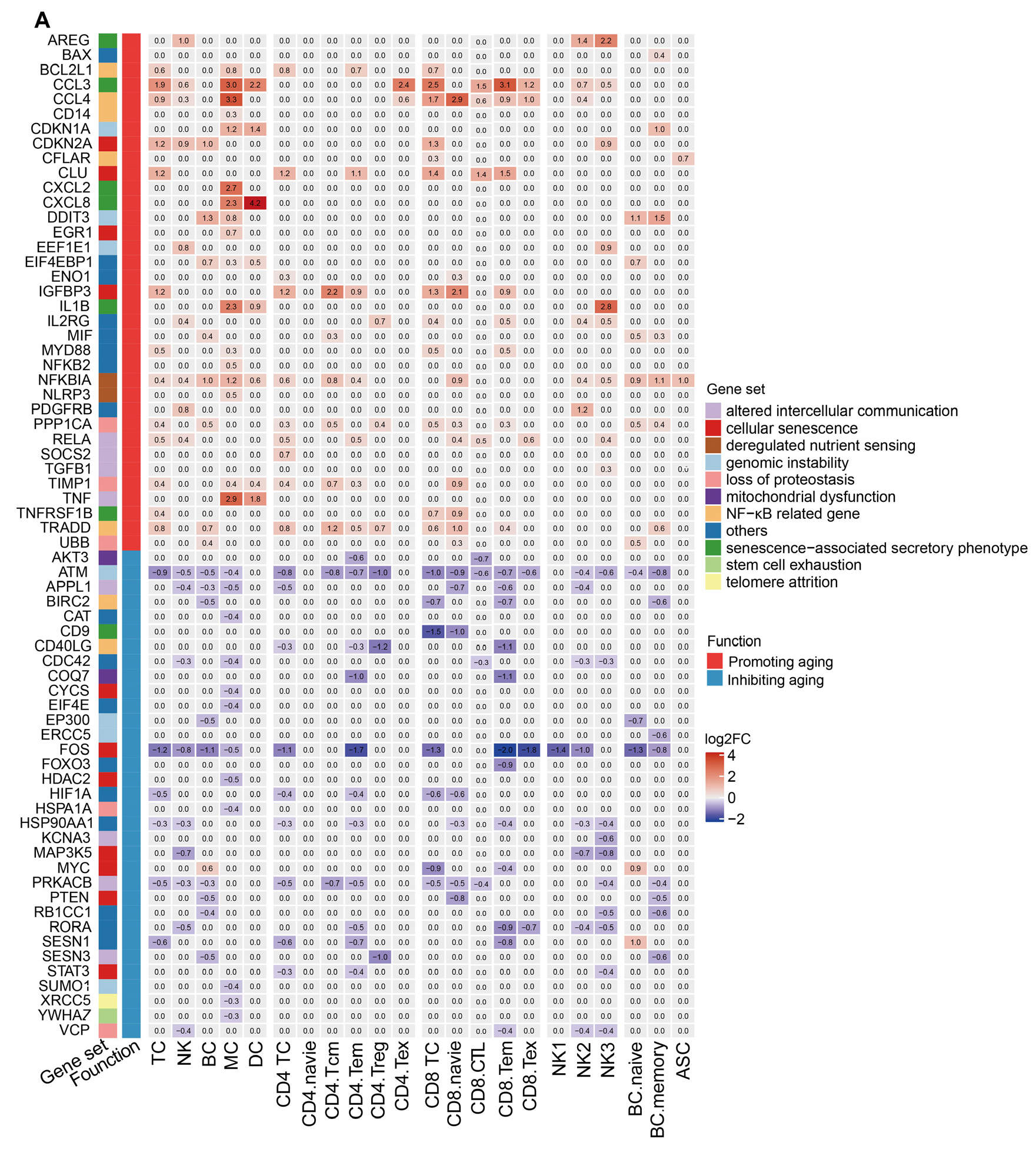


Fig. S3 | Immunocytes in the aging cohort exhibit distinctive age-associated molecular expression patterns.

**Supplementary Figure 4**

**
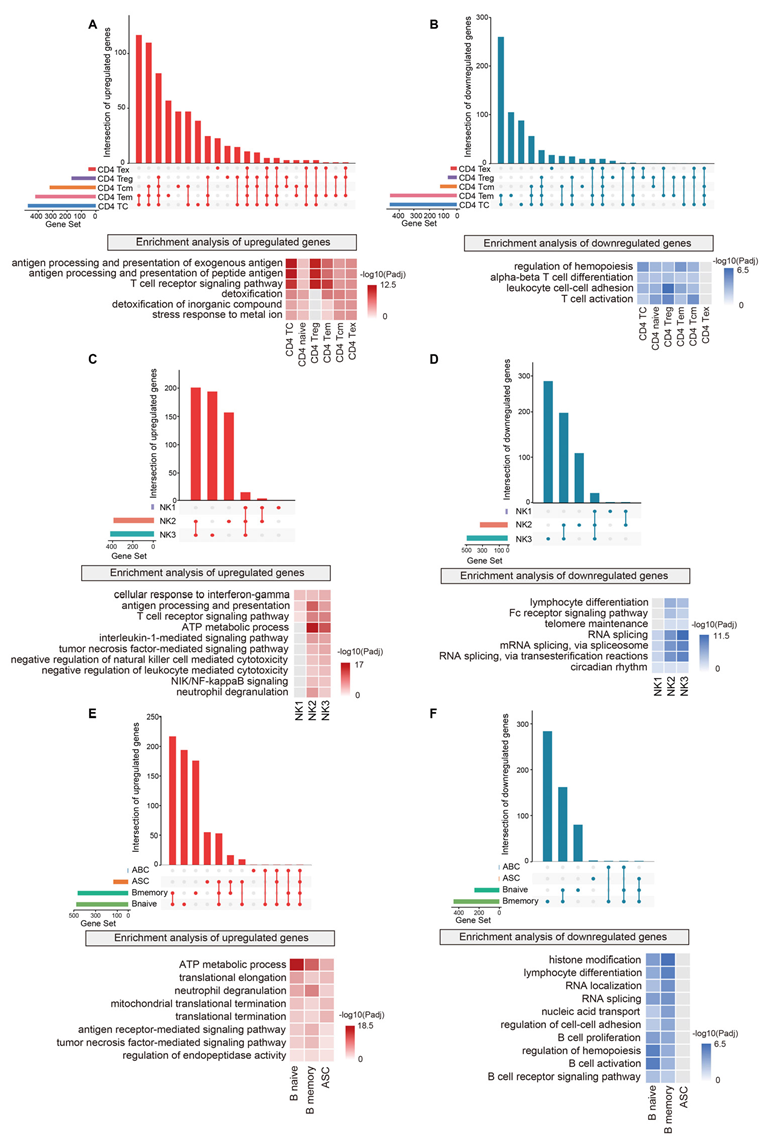
**

Fig. S4 | Differential analysis of transcriptomes and GO enrichment analysis for various immune cell subtypes. (A-B) Upregulated and downregulated genes along with their biologically enriched functions in different CD4 T cell subtypes during aging. (C-D) Upregulated and downregulated genes along with their biologically enriched functions in different NK cell subtypes during aging. (E-F) Upregulated and downregulated genes along with their biologically enriched functions in different B cell subtypes during aging. Note: Red and blue squares represent significantly upregulated and downregulated biological processes, respectively; gray squares represent processes that are not significantly enriched. The top 10 significantly enriched biological processes are visualized for each analysis.

**Supplementary Figure 5**

**
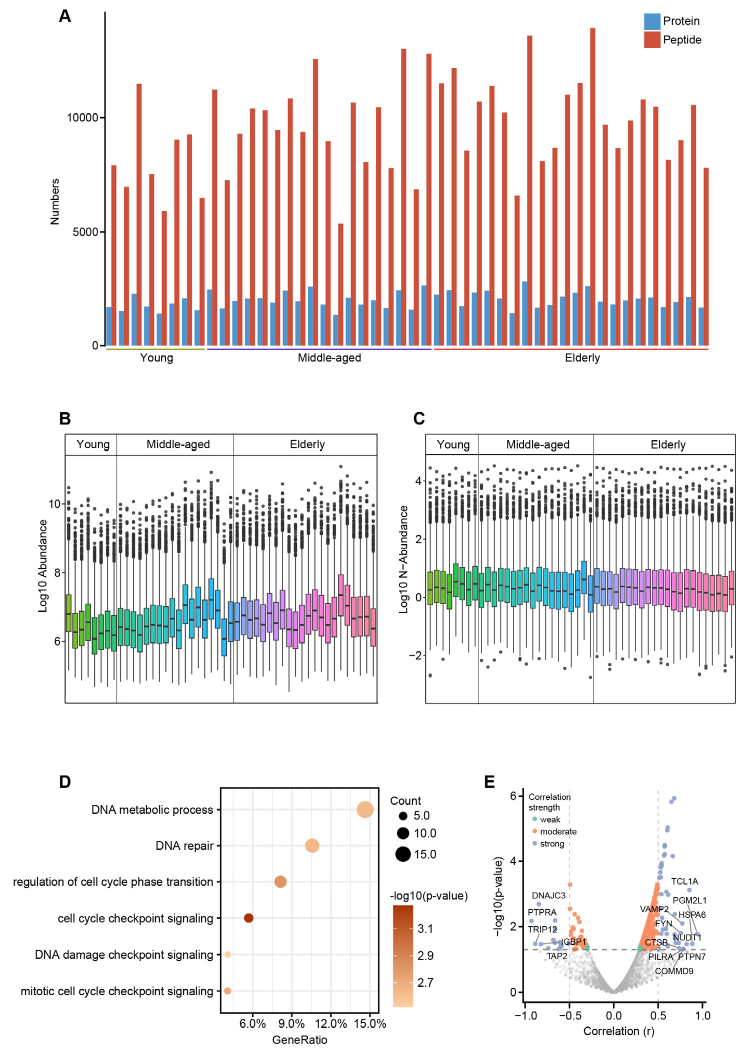
**

Fig. S5 | (A) Number of identified proteins and peptides in each sample across the three age groups. (B-C) Boxplots illustrating the distribution of raw protein quantification values and normalized protein quantification values for each sample. (D) GO Biological Process enrichment of 130 proteins identified exclusively in the elderly group. Dot size represents the number of genes mapped to each term; dot color indicates −log10(p-value). Terms are ordered by gene ratio (largest at top). (E) Volcano plot of per-gene mRNA–protein concordance. Each point represents one gene; the x-axis shows the Spearman correlation coefficient between mRNA and protein abundance and the y-axis shows the corresponding significance. Genes are categorized by correlation strength using absolute r: weak (0.1 ≤ |r| < 0.3), moderate (0.3 ≤ |r| < 0.5) and strong (|r| ≥ 0.5).

**Supplementary Figure 6**


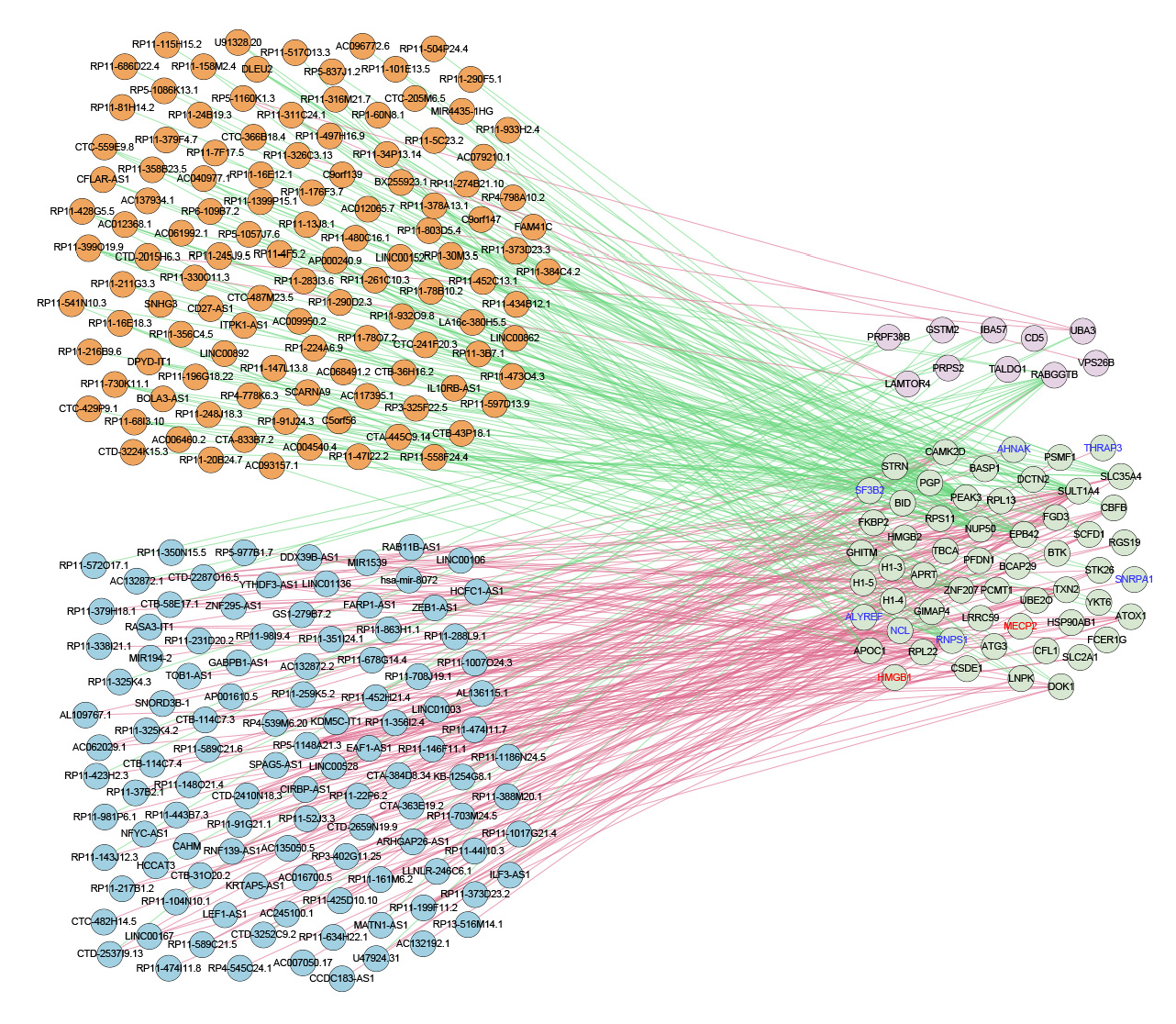


Fig. S6 | The association between the expression of age-associated lncRNAs and age- associated proteins. The orange and blue circles represent lncRNAs that are upregulated and downregulated with age, respectively. Similarly, the purple and green circles represent proteins that are upregulated and downregulated with age, respectively. The pink and green edges indicate positive and negative correlations, respectively. Splicing-related proteins are labeled in blue within the green circles, while proteins known to inhibit cellular senescence are labeled in red.

**Supplementary Figure 7**

**
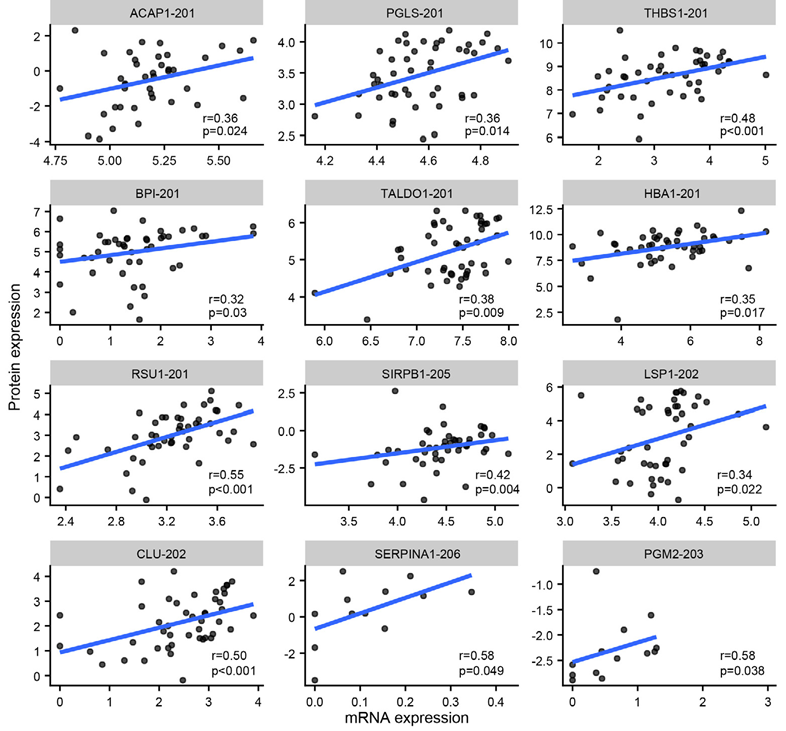
**

Fig. S7 | Transcript–protein correlations for 12 isoforms supported by isoform-unique peptides. Scatter plots show protein isoform intensities (log2-transformed, sample median-centered) versus transcript abundances (log2(TPM+1)) across 48 paired samples. Spearman correlation coefficient and nominal p-value are indicated in each panel.

**Supplementary Figure 8**


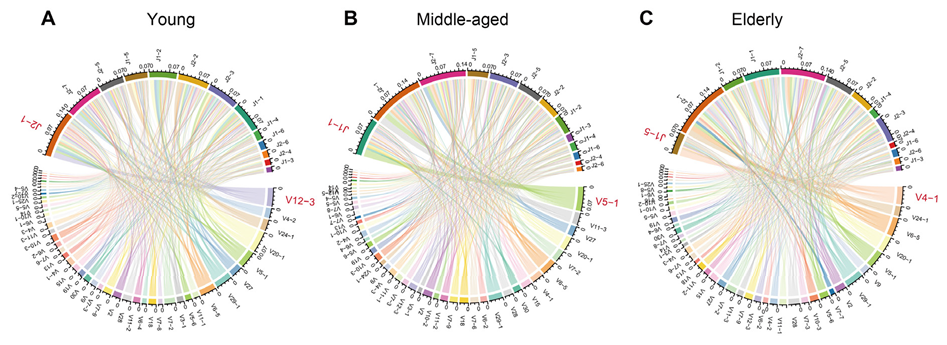


Fig. S8 | Circos plot illustrating the differences in the frequency of V and J gene usage among the three age groups. The width of the outer circular sectors represents the relative frequency of V or J genes, while the width of the connections between V and J gene combinations indicates their frequency of usage in specific age groups.

**Table S1. Information of each subject in the aging cohort**

**Table S2. Information of each subject in the Flow Cytometry cohort**

**Table S3. Age-associated lncRNAs**

**Table S4. Molecular Markers for 18 Subtypes of Immune Cells**

**Table S5. Age-associated proteins**

**Table S6. Correlation between age-associated lncRNAs and age-associated proteins**

**Table S7. Age-associated ASEs**

**Table S8. Correlation between ASEs of genes involved in protein regulation and the expression of protein profiles**

**Table S9. Sequences of primers used in TCRB library construction**
